# Fibrates Inhibit PLTP‐induced M2 Macrophage Infiltration and Increase the Sensitivity of Hepatocellular Carcinoma to ICIs

**DOI:** 10.1002/advs.202513257

**Published:** 2025-12-14

**Authors:** Xinyue Liang, Yaning Li, Jianxun Cai, Lisi Luo, Pengfei Yang, Yutong Chen, Jiancong Zhou, Yan Zeng, Jiaping Yu, Weiyu Zhang, Wenzheng Pang, Yufang Li, Chunhua Wen, Jian Li, Linjuan Zeng

**Affiliations:** ^1^ Cancer Center of the Fifth Affiliated Hospital Sun Yat‐sen University Zhuhai Guangdong 519000 China; ^2^ Guangdong Provincial Engineering Research Center of Molecular Imaging, the Fifth Affiliated Hospital Sun Yat‐sen University Zhuhai Guangdong 519000 China; ^3^ Guangdong‐Hong Kong‐Macao University Joint Laboratory of Interventional Medicine, The Fifth Affiliated Hospital Sun Yat‐sen University Zhuhai Guangdong 519000 China; ^4^ Department of Ultrasound, The Fifth Affiliated Hospital Sun Yat‐Sen University Zhuhai Guangdong 519000 China; ^5^ Department of Pathology, the Fifth Affiliated Hospital Sun Yat‐sen University Zhuhai Guangdong 519000 China; ^6^ Department of Oncology, Fujian Provincial Hospital Fuzhou University Affiliated Provincial Hospital Fuzhou Fujian 350001 China; ^7^ Department of Hematology, The Fifth Affiliated Hospital Sun Yat‐Sen University Zhuhai Guangdong 519000 China; ^8^ Department of Hepatobiliary Surgery, The Fifth Affiliated Hospital Sun Yat‐Sen University Zhuhai Guangdong 519000 China

**Keywords:** hepatocellular carcinoma, immune checkpoint inhibitors therapy, M2 macrophages, NF‐κB pathway, phospholipid transfer protein

## Abstract

High levels of M2 macrophages in the hepatocellular carcinoma (HCC) tumor microenvironment (TME) are associated with poor response to immune checkpoint inhibitors (ICIs). This study comprehensively investigated the role of phospholipid transfer protein (PLTP) in driving M2 macrophage polarization through bioinformatics, clinicopathological analysis, molecular docking, proteomics, biochemical and cellular assays. Additionally, strategies to enhance ICIs sensitivity are validated in multiple animal models. Results demonstrated that high M2 macrophage infiltration independently predicted inferior ICIs outcomes, and PLTP overexpression in HCC promoted M2 macrophage polarization. Mechanistically, PLTP bound to aurora kinase A (AURKA) and P65, forming a complex that induced P65 phosphorylation, thereby activating NF‐κB and upregulating IL‐6, IL‐8, and CSF‐1. Molecular docking revealed that GMB‐475 specifically bound to PLTP's functional domain (25‐245 AA), which competitively inhibited PLTP‐P65‐AURKA interactions and suppressing P65 phosphorylation. In vivo, GMB‐475 reduced M2 macrophage infiltration and suppressed tumor growth. Fibrates downregulated PLTP expression, decreased P65 phosphorylation, and synergized with ICIs in orthotopic and Myc‐driven HCC models. These findings highlight PLTP as a key mediator of M2 macrophage polarization via AURKA‐dependent NF‐κB activation. Targeting PLTP with inhibitor GMB‐475 or fibrates may improve ICIs efficacy, offering a promising therapeutic strategy for HCC.

## Graphical abstract

Phospholipid transfer protein(PLTP) plays a critical role in forming a complex with kinase A (AURKA) and P65. This interaction facilitates phosphorylation of P65 at Ser536, leading to the activation of the NF‐κB signaling pathway. Ultimately, this leads to the upregulation of downstream cytokines, including IL‐6, IL‐8, and CSF‐1, which promotes M2 macrophage polarization and contributes to the immunosuppressive tumor microenvironment.

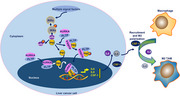

## 1 Introduction

Hepatocellular carcinoma (HCC) is the most common type of primary liver cancer and a major health concern worldwide.^[^ ^1^ ^]^ Lenvatinib, sorafenib, and chemotherapy have shown limited efficacy in prolonging the overall survival (OS) of patients with advanced HCC.^[^ ^2^ ^]^ Immune checkpoint inhibitors (ICIs), including anti‐PD‐1/PD‐L1 and Cytotoxic T lymphocyte associate protein‐4 (CTLA4) monoclonal antibodies, activate the immune system to effectively eliminate tumor cells and have shown promise in treating various types of tumors.^[^ ^3^, ^4^, ^5^, ^6^ ^]^ Based on the results of the IMbrave150 and ORIENT‐32 clinical trials, the combination of atezolizumab and bevacizumab as well as sintilimab and bevacizumab significantly prolonged progression‐free survival (PFS) and OS compared with traditional sorafenib monotherapy in patients with advanced‐stage HCC. They are recommended as the first‐line treatment options by the National Comprehensive Cancer Network (NCCN) guidelines. However, the overall response rate (ORR) remains low and most patients who initially benefit from treatment still face the risk of disease progression. Therefore, the efficacy of ICIs in patients with advanced HCC is hindered by the development of primary and secondary resistance.^[^ ^7^, ^8^ ^]^

Current evidence suggests that an immunosuppressive microenvironment contributes to resistance to immunotherapy. Macrophages are one of the most prevalent immune cells in the HCC microenvironment.^[^ ^9^, ^10^ ^]^ Macrophages may experience different polarization states depending on the signals they receive. Macrophages with the M2 phenotype usually exhibit pro‐carcinogenic properties and are thus called tumor‐associated macrophages (TAMs).^[^ ^11^ ^]^ Numerous studies have shown that an increase in the density of TAMs is often associated with poor prognosis for various cancers, including HCC.^[^ ^12^, ^13^, ^14^ ^]^ M2 macrophages play a crucial role in the immunosuppressive microenvironment of tumors.^[^ ^15^ ^]^ They form the core of the immunosuppressive cell and cytokine network, and the infiltrating macrophages in most tumors belong predominantly to the pro‐tumor M2 phenotype, which can also affect tumor cells by releasing cytokines or interacting directly, ultimately leading to drug resistance and tumor progression.^[^ ^16^, ^17^, ^18^ ^]^ M2 macrophages not only hinder cytotoxic T‐cell infiltration and induce T‐cell immunosuppression.^[^ ^19^, ^20^ ^]^ As such, they are important contributors to evasion of the tumor immune response. Therefore, blocking M2 macrophage recruitment and invasion is an emerging cancer treatment strategy. However, the mechanism of M2 macrophage infiltration in HCC remains largely unknown, and there are currently no effective interventions to reduce M2 macrophages in the tumor microenvironment (TME).

Phospholipid transfer protein (PLTP) is a lipid transporter that adopts a boomerang shape with N and C‐terminal barrel‐like structures linked together to create hydrophobic channels for lipid molecule transport.^[^ ^21^ ^]^ Their main function is to facilitate the exchange of amphiphilic lipids among circulating lipoproteins, cells, and tissues. This exchange significantly influences the plasma levels, turnover, and functions of very low‐density lipoprotein (VLDL), low‐density lipoprotein (LDL), and high‐density lipoprotein (HDL). In addition to its role in lipid metabolism, PLTP functions as an important inflammatory factor that participates in various physiological and pathological processes in the body. It is closely associated with cardiovascular diseases, neurodegenerative diseases, and adaptive and innate immunity.^[^ ^22^, ^23^ ^]^ PLTP deficiency has been shown to significantly decrease the size of atherosclerotic lesions, diminish monocyte/macrophage infiltration, and reduce the ability of LDL to induce chemotactic activity in monocytes.^[^ ^24^, ^25^ ^]^ Emerging evidence has indicated that PLTP plays a context‐dependent role in tumorigenesis. It has been implicated in lipid remodeling and signal transduction in cancers such as lung adenocarcinoma.^[^ ^26^, ^27^ ^]^ Recently large‐scale clinical proteomic studies have highlighted a significant association between elevated PLTP expression and increased HCC risk in high‐risk populations, suggesting its potential role as a biomarker and therapeutic target for liver cancer.^[^ ^28^ ^]^ Despite indications in the literature regarding the association between PLTP and poor prognosis in cancers,^[^ ^28^, ^29^, ^30^ ^]^ the precise effects of PLTP on the microenvironment and responses to ICIs in HCC remain to be explored.

In this study, we aimed to determine the key factors associated with M2 macrophage infiltration, identify effective interventions to target immunosuppressive TAMs in the HCC microenvironment, and ultimately develop potential therapeutic strategies to enhance HCC sensitivity to ICIs. Through comprehensive bioinformatics and clinicopathological analyses, PLTP was identified as a new biomarker related to M2 macrophage infiltration and poor response to ICIs therapy in patients with HCC. The mechanism of PLTP inducing M2 macrophage infiltration was observed both in vitro and in vivo, and chemical inhibition of PLTP expression was used to enhance the antitumor activity of the PD‐1 antibody in mice. Our study provides valuable insights into the role of PLTP in regulating TME and presents potential strategies for improving HCC immunotherapy.

## 2 Results

### 2.1 High Numbers of Tumor‐Infiltrating M2 Macrophages are Associated with Poor Prognosis in HCC

**Figure 1:**
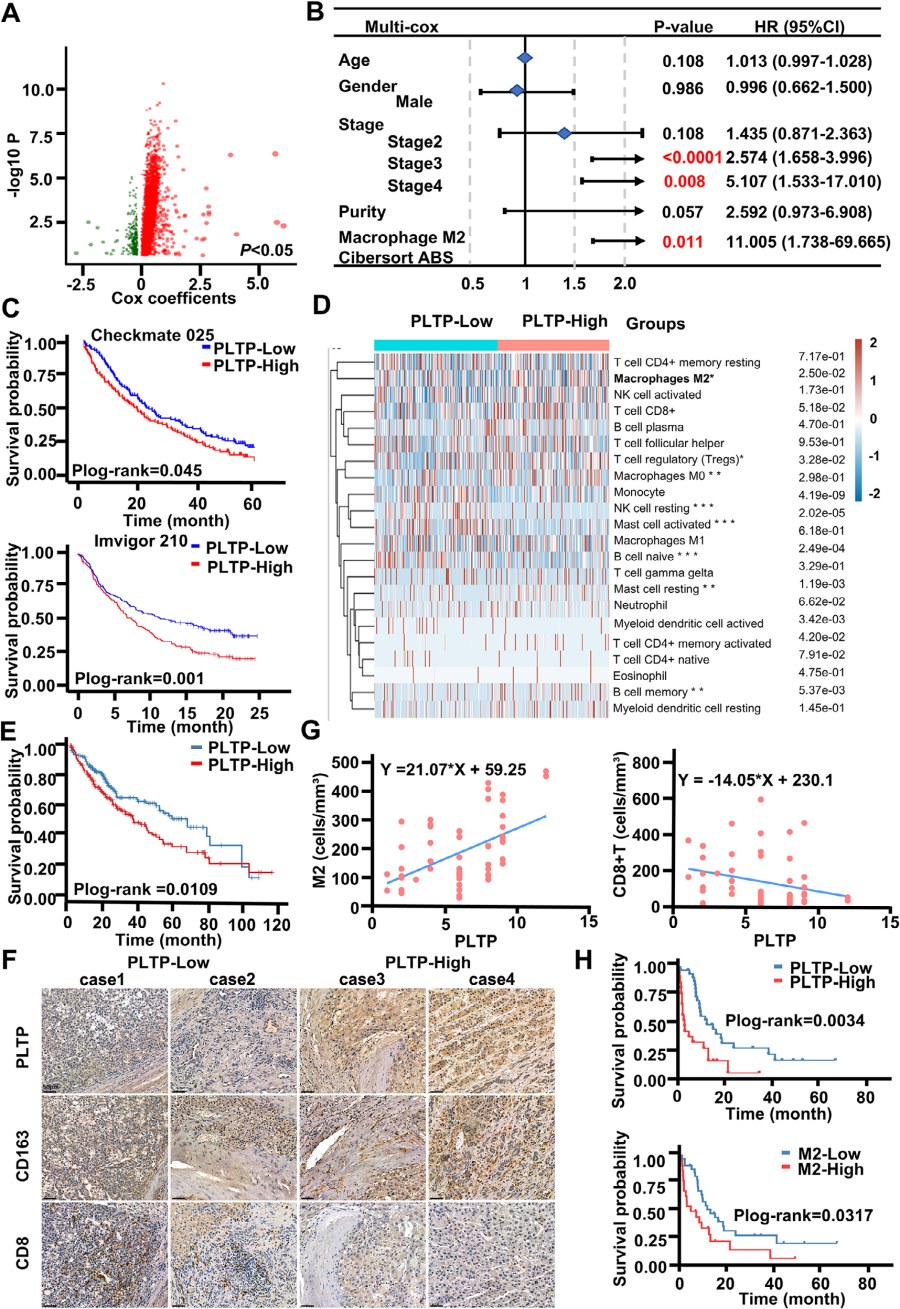
High numbers of tumor‐infiltrating M2 macrophages are associated with poor prognosis in HCC. A) Volcano plot of univariate Cox regression analysis in HCC (TCGA cohort). Red dots indicate genes associated with poor prognosis, while green dots indicate genes associated with favorable prognosis. B) Multivariate analysis reveals that M2 macrophage infiltration correlates with poor patient outcomes. C) Kaplan‐Meier analysis of overall survival in the immunotherapy‐treated cohort (Checkmate 025, ccRCC and Imvigor 210, bladder cancer), stratified by high and low PLTP expression levels. D) Heatmap of immune cell infiltration scores across samples; colors indicate relative expression levels. Statistical comparisons were performed using the Wilcoxon test for two‐group comparisons. *P < 0.05, **P < 0.01, ***P < 0.001. E) High PLTP expression is associated with poor prognosis in HCC patients from the TCGA‐LIHC cohort. F) Representative IHC images of PLTP, CD163, and CD8 expression in HCC tissues. Scale bar: 50 µm (magnification × 200). G) Correlation analysis of PLTP expression with CD163+ macrophages and CD8+T cells in HCC tissue specimens. H) PFS curves of 55 HCC patients treated with ICIs, stratified by high or low infiltration of PLTP‐expressing cells and CD163+ macrophages.

To evaluate the relationship between HCC progression and immune cell infiltration, we first performed univariate Cox analysis on TCGA‐LIHC data and then identified 4785 genes associated with prognosis, of which 4504 were linked to poor outcomes (**Figure** **1**A). Then, we intersected these genes with immune‐related genes from the ImmPort dataset and selected 176 immune genes for further analysis (Figure S1A, Supporting Information). Both univariate and Kaplan‐Meier analyses revealed that only M2 macrophage infiltration was significantly associated with a poor prognosis (Table S2; Figure S1B, Supporting Information). Multivariate Cox regression analysis confirmed that M2 macrophages (hazard ratio [HR] = 11.005, P = 0.011), Stage 3 (HR = 2.574, P < 0.0001), and Stage 4 (HR = 5.107, P = 0.008) were significantly associated with poor prognosis (Figure 1B).

Using TIMER2, we assessed the correlation between 176 immune genes and M2 macrophage infiltration, identifying 28 genes that were strongly correlated and 20 genes that were positively associated with M2 macrophage infiltration (Figure S2, Supporting Information). These 20 genes, including PLTP, may affect patient response to ICIs treatment by promoting M2 macrophage infiltration. We analyzed two immunotherapy cohorts (Checkmate 025, ccRCC, and Imvigor 210, bladder cancer) and found that only PLTP expression was significantly associated with overall survival (OS) in patients treated with nivolumab and atezolizumab (Figure 1C; Figure S3, Supporting Information). For HCC, TCGA‐LIHC data showed that high PLTP expression was correlated with increased M2 macrophage infiltration (Figure 1D) and poor prognosis (Figure 1E). Clinically, in the ICI‐treated cohort (n = 55), patients with higher PLTP expression in HCC tumor tissues exhibited increased M2 macrophage infiltration, reduced CD8+ T‐cell infiltration, and a worse prognosis (Figure 1F–H). These findings suggest that PLTP influences M2 macrophage infiltration and contributes to immunotherapy resistance in HCC.

### 2.2 PLTP Expression in HCC Cells Promotes Macrophage Recruitment and Polarization to M2 Macrophages Both in Vitro and in Vivo

**Figure 2:**
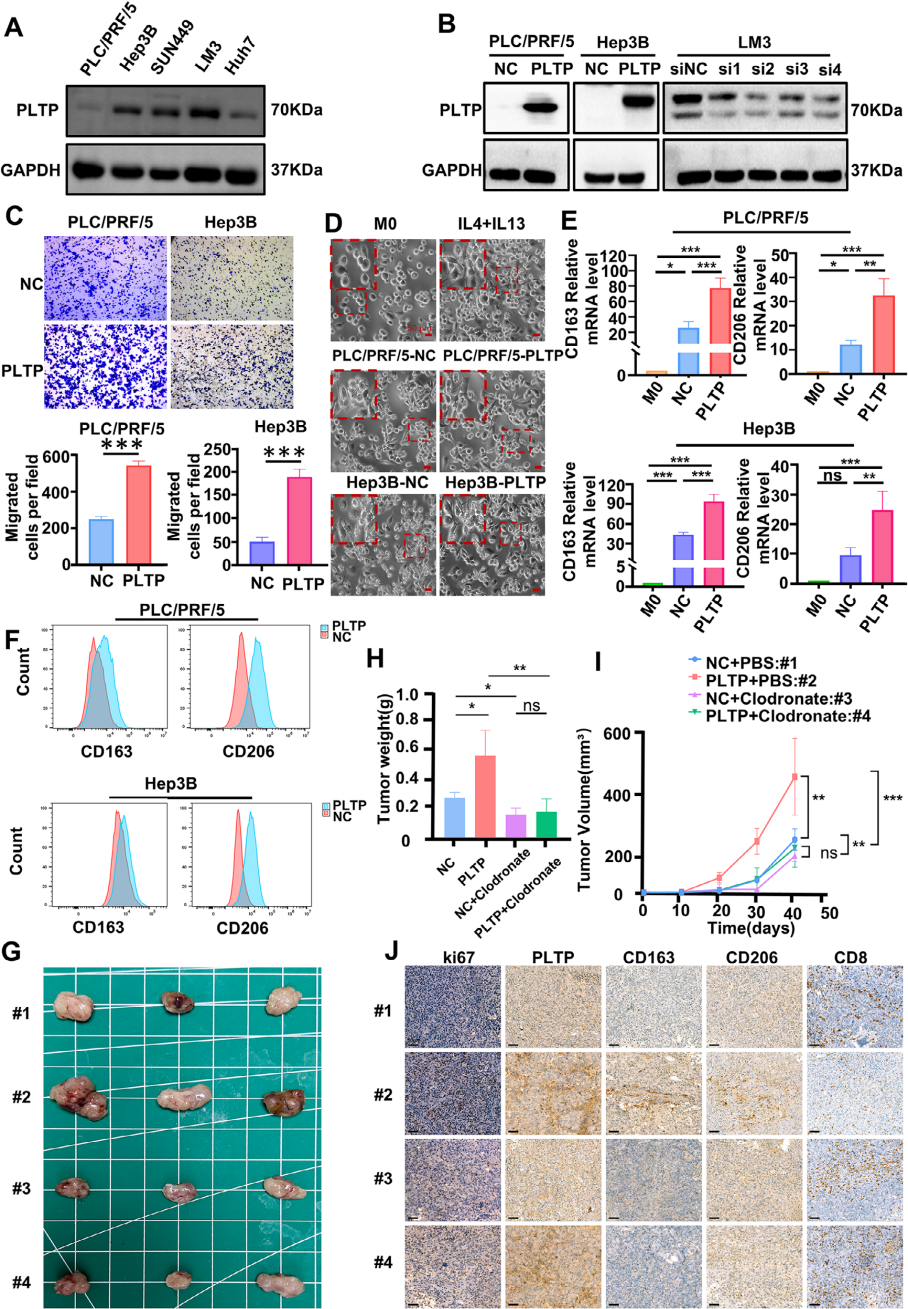
PLTP expression in HCC cells promotes macrophage recruitment and polarization to M2 macrophages. A) Basal expression levels of PLTP protein in various human HCC cell lines. B) PLTP protein expression in HCC cell lines following transfection with PLTP‐overexpressing plasmid or PLTP‐targeting siRNAs. Negative control (NC) plasmid or NC siRNA was used for comparison. C) Transwell migration assay evaluating the chemotactic effect of conditioned medium (CM) from HCC cells on THP‐1 monocyte migration. D) CM from PLTP‐overexpressing HCC cells induces morphological changes of M0 macrophages consistent with M2 macrophage polarization. E, F) qRT‐PCR and flow cytometry analyses showing increased expression of M2 macrophage markers CD163 and CD206 in macrophages co‐cultured with PLTP‐overexpressing HCC‐CM. G) Subcutaneous tumor growth in C57BL/6J mice treated with macrophage‐depleting agent clodronate or PBS as control. #1: NC+PBS; #2: PLTP+PBS; #3: NC+Clodronate; #4: PLTP+Clodronate. H, I) Comparison of tumor weights (H) and growth curves (I) across treatment groups. J) Representative IHC staining of Ki‐67, PLTP, CD163, CD206, and CD8 in mouse HCC tissues. Scale bar: 50 µm (magnification ×200). *P < 0.05, **P < 0.01, ***P < 0.001.

To better understand how PLTP expression in HCC cells affects immune microenvironment regulation, we first assessed PLTP expression in various human HCC cell lines (**Figure** **2**A). Based on the primary levels of PLTP, we selected PLC/PRF/5 and Hep3B cells to construct overexpression cell lines using the overexpression plasmid of PLTP (ov‐PLTP); cells transfected with the control plasmid (NC) served as a negative control. Alternatively, LM3, which has a relatively high level of PLTP expression, was used to construct knockdown cell lines using small interfering RNA targeting PLTP (si‐PLTP), with LM3 cells transfected with si‐NC serving as the control group (Figure 2B; Figure S4A, Supporting Information). Then, we observed the influence of PLTP expression on macrophage recruitment and polarization in HCC. THP‐1 cells were cocultured with conditioned media (CM) derived from HCC cells expressing varying levels of PLTP. As shown in Figure 2C, compared to the NC group, PLTP‐overexpressing PLC/PRF/5 and Hep3B cells induced more THP‐1 cells to migrate to the lower chamber. To investigate macrophage polarization induced by HCC, we induced the differentiation of THP‐1 monocytes into M0 macrophages by PMA treatment for 24 h. Then the culture medium was removed, and the supernatants from the HCC cells were added for co‐culture. M0 cells stimulated with IL‐4 and IL‐13 were used as positive controls for M2 macrophage polarization. M0 cells cocultured with PLTP‐overexpressing PLC/PRF/5 and Hep3B cell supernatants exhibited significant morphological changes such as elongated shapes (Figure 2D). In addition, the macrophages in the PLTP‐overexpressing coculture group exhibited a notable increase in the expression of CD163 and CD206 (Figure 2E,F; Figure S4B, Supporting Information). These findings suggest that PLTP expression in HCC may contribute to the promotion of macrophage recruitment and differentiation toward the M2 phenotype.

We further observed the effect of PLTP on the HCC immune microenvironment in mouse HCC allograft tumors. Lentivirus plasmids were used to construct PLTP‐overexpressing Hepa1‐6 cells (Hepa1‐6‐PLTP) and control cells (Hepa1‐6‐NC). First, Hepa1‐6‐NC and Hepa1‐6‐PLTP cells were injected into C57BL/6J mice to construct subcutaneous tumor models, and the Hepa1‐6‐PLTP group exhibited significantly enhanced tumor proliferation (Figure 2G, group #1 versus group #2). However, when macrophages were depleted by clodronate‐containing liposomes (Figure S4C, Supporting Information), clodronate treatment inhibited tumor growth compared with NC group (Figure 2G–I, group #3 versus group #1 and group #4 versus group #2) and the enhancement effect of PLTP overexpression on tumor growth was attenuated (Figure 2G, group #3 versus group #4 and Figure 2H,I). Pathological analysis showed that Hepa1‐6‐PLTP tumors have more M2 macrophages and fewer CD8+T cells in the stroma and a higher ki67 index compared to Hepa1‐6‐NC tumors (Figure 2J; Figure S4D, Supporting Information, group #1 versus group #2). After macrophage depletion, the number of infiltrated CD8+T cells increased and the tumor cell proliferation index decreased (group #3 versus group #1 and group #4 versus group #2, respectively). Importantly, no significant differences were observed between Hepa1‐6‐NC and Hepa1‐6‐PLTP (group #3 versus group #4). These results suggest that the promotional effect of PLTP on HCC tumors depends on M2 macrophage infiltration. These findings also support the hypothesis that high PLTP expression in HCC cells promotes the formation of an immunosuppressive microenvironment, which could potentially impair the efficacy of ICIs.

### 2.3 PLTP Promotes the Infiltration and Polarization of M2 Macrophages by Activating HCC NF‐κB Signal Pathway

**Figure 3:**
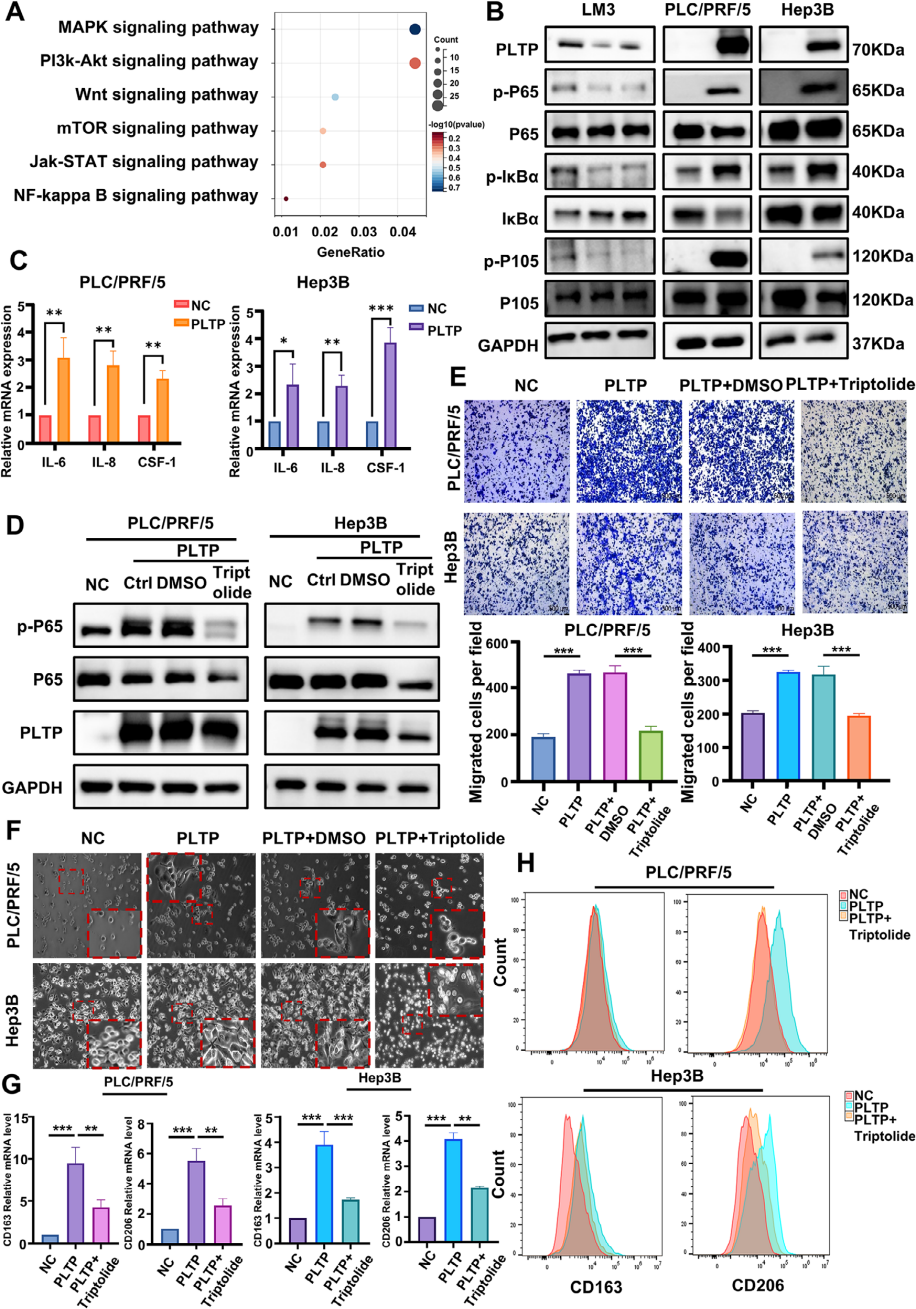
PLTP promotes the infiltration and polarization of M2 macrophages by activating HCC NF‐κB signal pathway. A) Bubble plot showing KEGG pathway enrichment of genes upregulated by PLTP overexpression. B) Western blot analysis of PLTP, phospho‐P65 (p‐P65), phospho‐IκBα (p‐IκBα), and phospho‐P105 (p‐P105) in HCC cells following PLTP overexpression or knockdown. C) qRT‐PCR analysis of IL‐6, IL‐8, and CSF‐1 mRNA expression following PLTP overexpression. D) The NF‐κB pathway inhibitor triptolide suppresses PLTP‐induced activation of P65 in PLC/PRF/5 and Hep3B cells. E) Transwell migration assay showing that PLTP‐induced recruitment of THP‐1 cells is attenuated by triptolide treatment. F) Microscopic analysis revealing that triptolide blocks the morphological polarization of M0 macrophages toward the M2 phenotype induced by PLTP‐HCC conditioned medium (CM). G) qRT‐PCR analysis demonstrating upregulation of CD163 and CD206 in M0 macrophages co‐cultured with PLTP‐HCC CM, which is reversed by triptolide. H) Flow cytometry analysis showing increased numbers of CD163+ and CD206+ macrophages after co‐culture with PLTP‐HCC CM, which is inhibited by triptolide. *P < 0.05, **P < 0.01, ***P < 0.001.

Tumor‐associated inflammatory responses are often closely linked to the immunosuppressive microenvironment. Various inflammatory mediators (such as IL‐4, IL‐10, IL‐13) and tumor‐associated factors (such as CSF‐1, TGF‐β) can promote the polarization of macrophages to the M2 phenotype. In addition to being a key factor in lipid metabolism, PLTP is an important inflammatory molecule involved in various physiological and pathological processes. We hypothesized that PLTP promotes M2 macrophage polarization by activating inflammatory pathways. We performed RNA sequencing (RNA‐seq) to analyze the gene profiles of Hep3B cells overexpressing NC or PLTP. Indeed, several inflammatory pathways were enriched in genes differentially expressed in Hep3B‐PLTP compared to Hep3B‐NC (**Figure** **3**A). As validated by western blot assay, only the NF‐κB pathway exhibited significant activation, accompanied by increased phosphorylation levels of key proteins such as P65, IκBα, and P105 (Figure 3B; Figure S5A, Supporting Information). The NF‐κB signaling pathway is crucial in intercellular information transmission, playing a core role in regulating immune response, inflammation, cell growth, and death among various biological processes. NF‐κB transcriptionally upregulates various cell inflammatory factors (such as IL‐6, IL‐8, IL‐1β, etc.), creating an inflammatory microenvironment that not only promotes tumor metastasis but also diminishes the effectiveness of immunotherapy.

We examined the mRNA levels of downstream target genes of NF‐κB, including IL‐6, IL‐8, and CSF‐1, which are potent chemokines promoting M2 macrophage polarization, and found significantly increased expression levels in the PLTP overexpression group compared to the NC group (Figure 3C). When the NF‐κB inhibitor, triptolide, was added into the culture medium containing PLC/PRF/5‐PLTP and Hep3B‐PLTP cells, the expression levels of P65 and phosphorylated P65 (p‐P65) decreased markedly (Figure 3D), indicating that triptolide inhibited PLTP‐induced NF‐κB pathway activation. Consequently, triptolide inhibited THP‐1 recruitment in the PLTP overexpression supernatant (Figure 3E) and suppressed the polarization of M0 macrophages into M2 macrophages compared to the control group treated with DMSO (Figure 3F–H; Figure S5B, Supporting Information). To further investigate whether the role of PLTP in macrophage recruitment and polarization depends on P65, we knocked down P65 expression in HCC cells using specific siRNA. Western blot analysis confirmed a significant decrease in P65 protein levels compared to the NC group (Figure S5C, Supporting Information). Under these conditions, we found that overexpression of PLTP failed to enhance the recruitment of THP‐1 cells in Transwell migration assays (Figure S5D, Supporting Information). Moreover, the proportion of M2‐type macrophages, as indicated by expression of CD206 and CD163, did not increase after PLTP overexpression in the P65‐knockdown setting (Figure S5E–G, Supporting Information). These findings indicate that PLTP promotes the infiltration and polarization of M2 macrophages relies on activation of HCC NF‐κB signal pathway.

### 2.4 PLTP Binds to P65 and Cooperates with Aurora Kinase A(AURKA) to Promote P65 Nuclear Translocation and NF‐κB Activation

**Figure 4:**
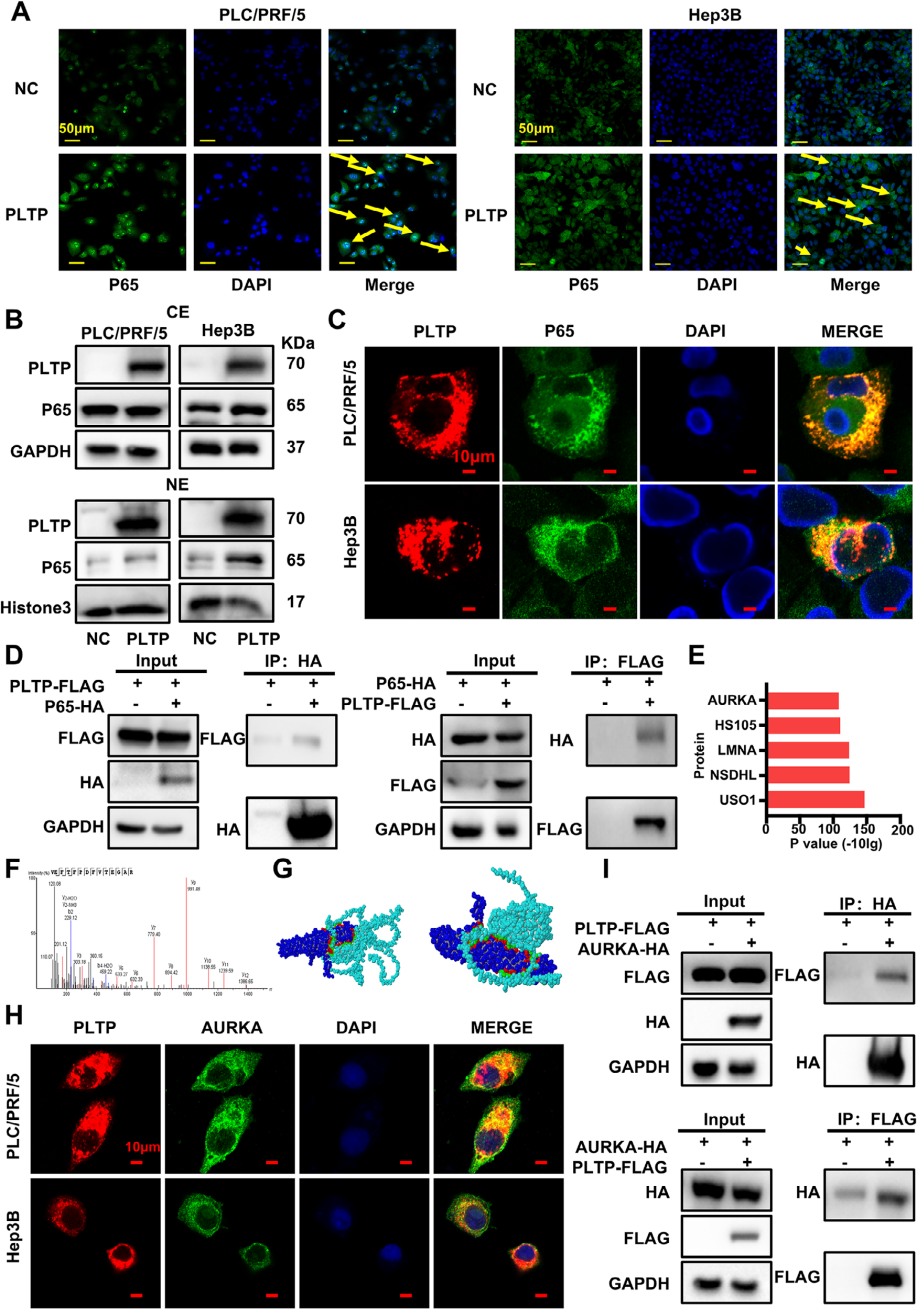
PLTP binds to P65 and cooperates with AURKA to promote P65 nuclear translocation and NF‐κB activation. A) Immunofluorescence analysis showing that PLTP overexpression promotes nuclear translocation of P65. Scale bar: 50 µm. B) Western blot analysis revealing increased nuclear levels of P65 upon PLTP overexpression. C) Immunofluorescence co‐localization analysis showing subcellular co‐localization of PLTP and P65. Scale bar: 10 µm. D) Co‐immunoprecipitation (Co‐IP) assay confirming the interaction between PLTP and P65. E) Mass spectrometry analysis identifying the top five candidate proteins potentially interacting with PLTP. F) Chromatographic profile of AURKA identified by mass spectrometry. G) The 3D spatial pattern of PLTP combined with P65 and AURKA, blue representing PLTP, green representing P65 (left) and AURKA (right), and red indicating the binding regions. H) Immunofluorescence analysis showing co‐localization of PLTP and AURKA. Scale bar: 10 µm. I) Co‐IP assay confirming the interaction between PLTP and AURKA.

When cells are in an inactive state, P65 is located in the cytoplasm and binds to the inhibitor protein IκBα. Upon receiving stimuli signals, IκBα is phosphorylated and then degraded, thus releasing P65. Subsequently, free P65 translocates from the cytoplasm to the nucleus, during this process, P65 is phosphorylated to p‐P65, and initiates the transcriptional regulation of downstream target genes. Immunofluorescence confirmed that more P65 was translocated into the nucleus in PLTP‐HCC cells (**Figure** **4**A). To elucidate the regulatory mechanisms underlying PLTP‐induced P65 phosphorylation, we extracted the nuclear and cytoplasmic proteins from PLC/PRF/5 and Hep3B cells. Western blot analysis revealed that both PLTP and P65 protein levels increased in the nuclear fraction following PLTP overexpression (Figure 4B). Immunofluorescence analysis further verified the co‐localization of PLTP and P65 within the cells, both in the plasma and nucleus (Figure 4C). Co‐immunoprecipitation (Co‐IP) experiments confirmed the binding of PLTP to P65 (Figure 4D). In addition, we found that increased PLTP reduces the binding between P65 and IκBα. Therefore, PLTP can not only directly bind to free P65 to form a PLTP‐P65 complex, but also competitively inhibit the formation of P65‐IκBα complex. (Figure S5H, Supporting Information). Based on these findings, we hypothesized that PLTP binds and activates P65 and ultimately leading to the activation of the NF‐κB pathway.

The mechanism by which PLTP‐P65 facilitates P65 phosphorylation needs to be elucidated. The proteins precipitated by immunoprecipitation with a PLTP antibody were analyzed by liquid chromatography‐tandem mass spectrometry (LC‐MS/MS). Among the top five PLTP‐interacting proteins, Aurora kinase A (AURKA) was notably upregulated in tumor tissues and was significantly associated with poor overall survival, suggesting its potential functional relevance in PLTP‐mediated tumor progression (Figure 4E; Figure S6A, Supporting Information) (HS105 cannot be found). AURKA is a serine/threonine kinase involved in the regulation of cell division and is frequently upregulated in various human cancers (Figure 4F). We performed a protein docking analysis using GRAMM to model the interactions between PLTP and P65, AURKA. Figure 4G exhibited the three‐dimensional spatial pattern of the PLTP combined with P65 and AURKA. The colocalization of PLTP and AURKA was confirmed by immunofluorescence staining (Figure 4H), and their binding was further validated by Co‐IP (Figure 4I). Interestingly, no binding was observed between P65 and AURKA (Figure S6B, Supporting Information).

These findings indicate that PLTP facilitates NF‐κB pathway activation by promoting P65 phosphorylation and nuclear localization, potentially through a mechanism involving AURKA. The PLTP‐P65‐AURKA axis may represent a novel regulatory pathway for HCC progression.

### 2.5 PLTP Functions as an Intermediary for AURKA‐Mediated P65 Phosphorylation and can be Targeted by GMB‐475

**Figure 5:**
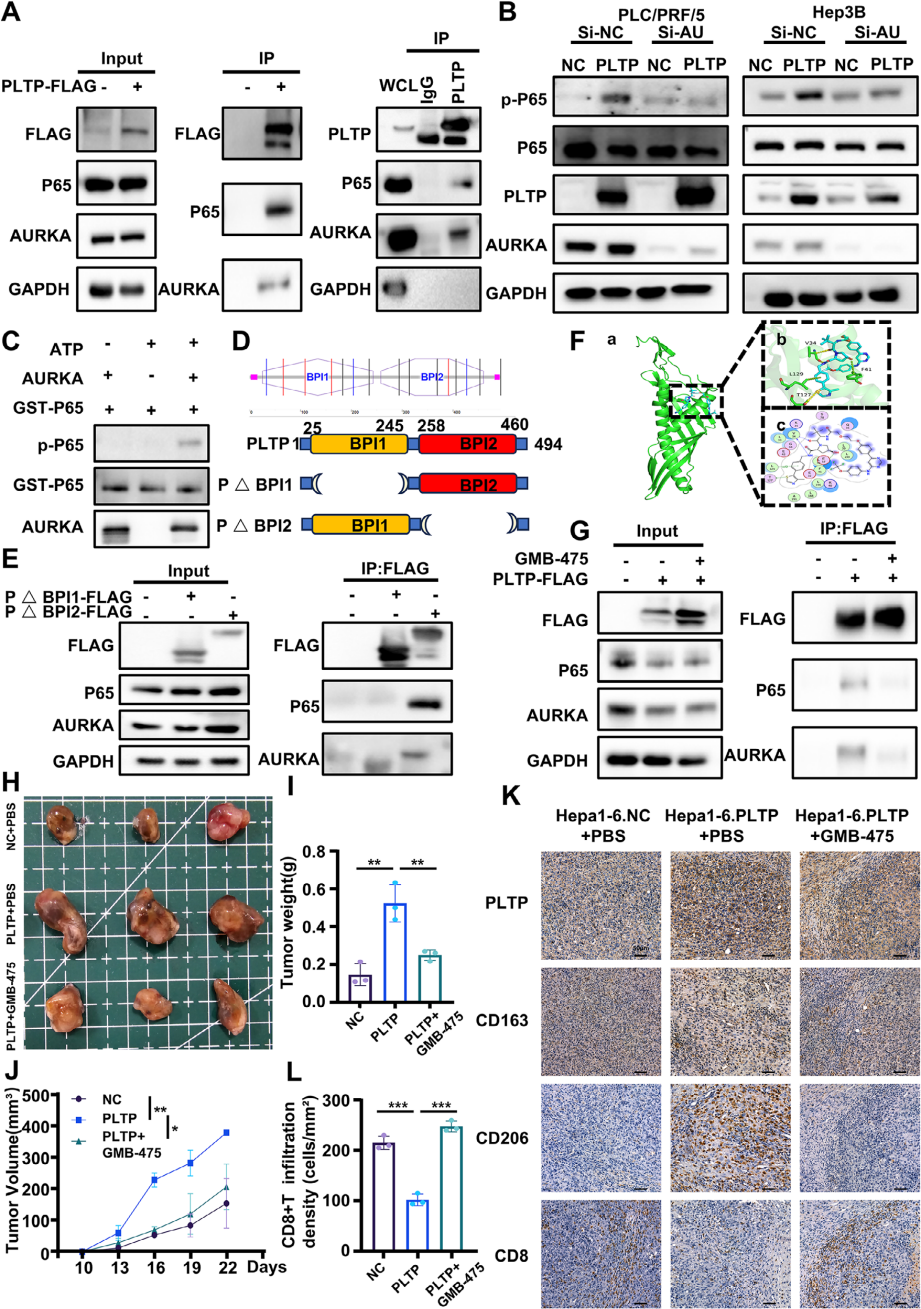
PLTP functions as an intermediary for AURKA‐mediated P65 phosphorylation and can be targeted by GMB‐475. A) Exogenous and endogenous Co‐IP assays show that PLTP simultaneously interacts with both AURKA and P65. B) Knockdown of AURKA abolishes PLTP‐induced phosphorylation of P65. C) In vitro kinase assay demonstrating that P65 is a direct phosphorylation substrate of AURKA. D) Schematic of PLTP functional domain prediction by the SMART database and the design of domain‐specific PLTP truncation plasmids. E) Co‐IP experiments identifying the binding domains of PLTP responsible for interaction with AURKA and P65. F) Molecular docking analysis of GMB‐475 with PLTP: (a) Overall 3D view showing GMB‐475 with carbon atoms in magenta, oxygen in red, nitrogen in blue, and the protein in green cartoon representation. (b) Enlarged 3D view showing interactions between GMB‐475 and surrounding amino acid residues. Yellow dashed lines represent hydrogen bonds, red dashed lines indicate ionic bonds, and dashed purple lines indicate aromatic ring–hydrogen stacking. (c) 2D interaction map indicating hydrogen bonds (arrows) and aromatic stacking (dashed lines). G) Co‐IP assays demonstrate that GMB‐475 disrupts the interaction of PLTP with both AURKA and P65. H) Representative tumor images from different treatment groups: Hepa1‐6‐NC + PBS; Hepa1‐6‐PLTP + PBS; Hepa1‐6‐PLTP + GMB‐475. I, J) Tumor weight (I) and growth rate (J) comparisons across treatment groups. K) Representative IHC images of tumor sections showing reduced M2 macrophage infiltration (CD163+, CD206+) and enhanced CD8+ T‐cell infiltration following GMB‐475 treatment. Scale bar: 50 µm (magnification ×200). L) Quantification of CD8+ cells by IHC in subcutaneous tumor tissues from C57BL/6J mice. *P < 0.05, **P < 0.01, ***P < 0.001.

Based on these observations, we hypothesized that PLTP serves as an intermediary between P65 and AURKA, thereby facilitating P65 phosphorylation by AURKA. To verify this hypothesis, we performed both endogenous and exogenous Co‐IP experiments to confirm that PLTP could bind simultaneously to both P65 and AURKA (**Figure** **5**A; Figure S6C, Supporting Information), supporting its role as a bridging molecule within the triple factor compound PLTP‐P65‐AURKA.

According to large‐scale phosphoproteomic databases such as PhosphoSitePlus, ser276 and ser536 are among the most extensively studied and functionally significant phosphorylation sites on P65, with abundant literature documenting their roles in NF‐κB regulation.^[^ ^31^ ^]^ (Figure S6D, Supporting Information). First, we examined the phosphorylation levels of P65 at ser276 and ser536 by overexpressing PLTP. We found that PLTP did not affect the phosphorylation of P65 at ser276 (Figure S6E, Supporting Information) but increased its phosphorylation at the ser536 site. To further validate this mechanism, AURKA was silenced in HCC cells using siRNA. AURKA knockdown significantly reversed PLTP‐mediated phosphorylation of P65‐ser536 (Figure 5B). In vitro kinase assays revealed that AURKA directly phosphorylates P65 in the presence of ATP, establishing P65 as a direct substrate of AURKA for the first time (Figure 5C). Notably, this phosphorylation event was independent of the PLTP phospholipid transfer activity. This is evidenced by the fact that the phospholipid transfer‐deficient PLTP mutant (M159E) ^[^ ^32^ ^]^ did not alter p‐P65 levels. In contrast, siRNA‐mediated knockdown of AUKRA markedly suppresses P65 phosphorylation induced by both wild‐type PLTP and the M159E mutant (Figure S7A, Supporting Information).

We used the SMART database to identify two functional regions of PLTP: BPI1 (25‐245 aa) and BPI2 (258‐460 aa) (Figure 5D). Truncated plasmids, P △ BPI1 (lacking the BPI1 region) and P △ BPI2 (lacking the BPI2 region), were constructed (Figure 5D). Co‐IP assays showed that expression of P △ BPI1 failed to bind to either P65 or AURKA, while P △ BPI2 still bound to both, indicating that the BPI1 region is critical for PLTP binding (Figure 5E).

Subsequently, we focused on the PLTP 25‐245 domain for virtual screening to identify potential inhibitors that could disrupt the PLTP‐P65 or PLTP‐AURKA interactions. Structural analysis revealed a distinct binding site for 1,2‐diacyl‐sn‐glycerol‐3‐phosphocholine (Figure 5F). Several ligands were screened for their ability to combine with the PLTP‐BPI1. Among the top five compounds with the highest binding affinities, GMB‐475 (c2) effectively inhibited PLTP overexpression‐induced P65 phosphorylation (Figure 5F; Figure S7B, Table S3, Supporting Information). To determine whether GMB‐475 affects the stability of PLTP, AURKA, or P65, we treated HCC cells with increasing concentrations of GMB‐475 for various durations. Western blot analysis revealed no significant changes in the protein levels of PLTP, AURKA, or P65, even at higher concentrations or extended treatment times, indicating that GMB‐475 did not induce target degradation (Figure S7C, Supporting Information). To assess the direct binding between GMB‐475 and PLTP, we performed a Cellular Thermal Shift Assay (CETSA). Marked thermal stabilization of PLTP was observed in GMB‐475 treated cells compared to the DMSO control group, demonstrating the direct engagement of GMB‐475 with PLTP (Figure S7D, Supporting Information). However, this thermal stabilization effect was abolished in HCC cells with the △BPI1 mutant of PLTP, indicating that GMB‐475 fails to stabilize PLTP when its specific BPI1 domain is deleted (Figure S7D, Supporting Information).  Co‐IP experiments confirmed that GMB‐475 significantly disrupted the interactions between PLTP and P65, or PLTP and AURKA (Figure 5G). Additionally, we reconstituted PLTP‐P65‐AURKA interactions in a cell‐free system using purified recombinant proteins. Pull‐down assays showed that GMB‐475 directly disrupted these protein‐protein interactions (Figure S7E, Supporting Information). The therapeutic potential of GMB‐475 was further assessed in C57BL/6J mice bearing Hepa1‐6 subcutaneous tumors. As shown in Figure 5H–J, PLTP overexpression promoted tumor growth, whereas tumors in the Hepa1‐6‐PLTP + GMB‐475 group were significantly smaller than those in the Hepa1‐6‐PLTP + PBS group, indicating that GMB‐475 treatment abrogated the tumor‐promoting effects of PLTP. IHC revealed that GMB‐475 decreased the number of M2 macrophages and increased CD8+T cell infiltration in the TME (Figure 5K,L).

Collectively, our results demonstrate that PLTP binds to P65 and AURKA together and mediates P65 phosphorylation by AURKA. P65 is a direct substrate of AURKA, and was discovered for the first time in this study. Furthermore, the functional region within 25‐245 amino acid is critical for the formation of PLTP‐P65‐AURKA. GMB‐475 effectively targeted the 25‐245 amino acids domain of PLTP, thereby disrupting the PLTP‐P65‐AURKA complex formation. Consequently, GMB‐475 exerted potent activity in the remodeling of the tumor immune microenvironment.

### 2.6 Fenofibrates Reverse the Inhibitory Effect of PLTP on Anti‐PD‐1 Monoclonal Antibody Therapy

**Figure 6:**
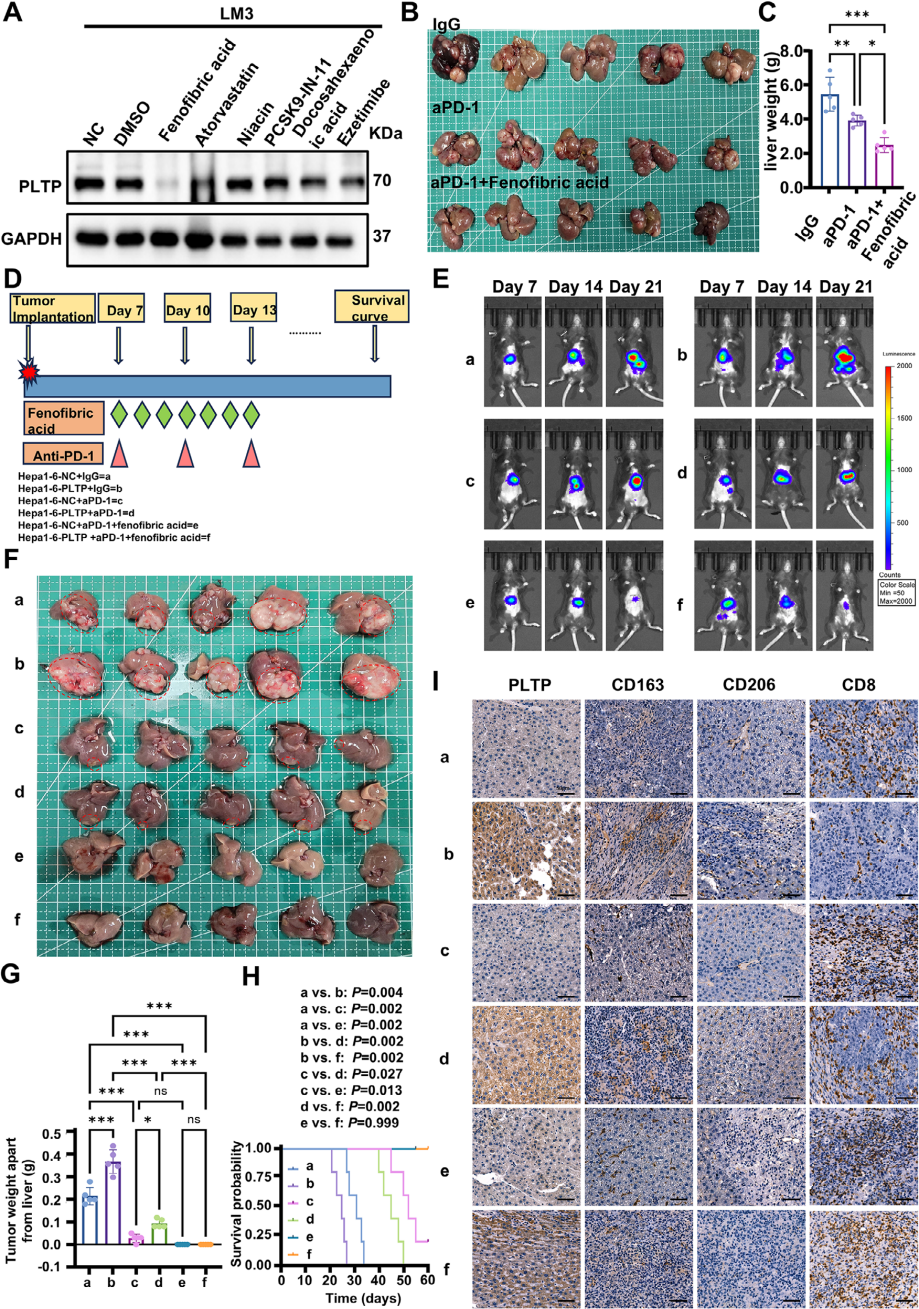
Fenofibrates reverse the inhibitory effect of PLTP on anti‐PD‐1 therapy. A) Western blot analysis was performed to assess the endogenous expression levels of PLTP in LM3 cells following treatment with six antilipemic drugs. B) Representative image of tumor harvested from Alb‐Cre⁺/MYC⁺ mice with IgG, aPD‐1, and aPD‐1 + fenofibric acid therapy. C) Comparison of liver weights among different groups. D) Workflow for treating orthotopic HCC tumors with anti‐PD‐1 and fenofibric acid. (a: NC+IgG; b: PLTP+IgG; c: NC+aPD‐1; d: PLTP+aPD‐1; e: NC+aPD‐1+fenofibric acid; f: PLTP+aPD‐1+fenofibric acid) E) Representative bioluminescence imaging on days 7, 14, and 21 in orthotopic HCC model mice subjected to the indicated treatments. F) Representative image of Hepa1‐6 cell‐derived tumor harvested from C57BL/6J mice on day 21. G) Comparison of tumor weights among different groups. H) Kaplan‐Meier survival curves for orthotopic HCC mice. I) Representative images of PLTP, CD163, CD206, and CD8 IHC staining in mouse HCC tissues. Scale bar: 50 µm (magnification ×200). *P < 0.05, **P < 0.01, ***P < 0.001.

Data compiled from databases, clinical trials, and clinical pathological analyses, demonstrate that PLTP is a negative prognostic factor in patients with HCC receiving ICIs treatment. A potential mechanism underlying ICIs resistance is that high PLTP expression in HCC cells promotes M2 macrophage infiltration. However, there are currently no validated PLTP inhibitors. Given that PLTP is an important lipoprotein involved in lipid metabolism and is closely associated with atherosclerosis, we hypothesized that antilipidemic drugs might lower PLTP levels, thereby potentially enhancing the efficacy of immunotherapy in HCC. First, we selected six commonly used antilipemic drugs, including fibrates, statins, niacin, PCSK9 inhibitors, omega‐3 fatty acids, and ezetimibe, and added them to the LM3 cell culture medium. As shown in **Figure** **6**A, the fibrates markedly reduced endogenous PLTP levels in LM3 cells. In addition, fibrates inhibited the phosphorylation of P65 (Figure S8A, Supporting Information). Accordingly, the treatment of LM3 cells with fibrates significantly impaired their ability to recruit macrophages and induce M2 polarization, as demonstrated in Figure S8B and C (Supporting Information). In addition, the mRNA levels of key macrophage‐recruiting cytokines, including IL‐6, IL‐8, and CSF‐1, were markedly reduced following fibrate treatment (Figure S8D, Supporting Information). To establish a spontaneous hepatocellular carcinoma model, H11‐LSL‐Myc mice were crossed with liver‐specific Cre‐expressing mice (Alb‐Cre), generating Alb‐Cre⁺/MYC⁺ offspring. To evaluate the role of fenofibric acid in ICIs therapy, Alb‐Cre⁺/MYC⁺ mice were randomly assigned to 3 treatment groups (n = 5): (i) immunoglobulin G (IgG), (ii) anti–PD‐1 antibody (aPD‐1), and (iii) aPD‐1 + fenofibric acid. As shown in Figure 6B,C, the combination therapy significantly suppressed tumor growth compared to treatment with anti‐PD‐1 alone. This was accompanied by increased CD8+T cell infiltration and reduced M2 macrophage density (Figure S8E, Supporting Information). We further investigated whether fibrates could reduce HCC PLTP levels and enhance the anti‐tumor efficiency of the PD‐1 antibody in vivo using C57BL/6J mice bearing Hepa1‐6‐NC or Hepa1‐6‐PLTP‐HCC orthotopic tumors (15 mice/group). Mice in each group (NC or PLTP) were further divided into three treatment subgroups (five mice/group) receiving IgG, aPD‐1, or aPD‐1 + fenofibric acid treatment. The dosing schedule is shown in Figure 6D.

Bioluminescence imaging revealed detectable tumor signals as early as day 7 following orthotopic HCC implantation, with no significant differences in luminescence intensity observed among the treatment groups at this time point. However, on days 14 and 21 post‐implantation, mice receiving the combination of aPD‐1+ fenofibric acid (groups e and f) exhibited significantly reduced luminescence intensity in orthotopic HCC lesions compared with those treated with anti‐PD‐1 monotherapy (groups c and d) or IgG control (groups a and b). Notably, in the anti‐PD‐1 monotherapy group, tumors in PLTP‐overexpressing (PLTP) mice (group d) showed higher luminescence intensity than those in the negative control (NC) group (group c). In contrast, in the combination treatment group (aPD‐1+ fenofibric acid), no significant difference in luminescence intensity was observed between PLTP (group f) and NC mice (group e), suggesting that fenofibric acid effectively overcame PLTP‐mediated resistance to immunotherapy (Figure 6E).

Pathological analysis further confirmed that fenofibric acid enhanced the antitumor activity of aPD‐1. As shown in Figure 6F,G, the tumors in the PLTP group (group b) were significantly larger than those in the NC group (group a). Anti‐PD‐1 monotherapy effectively inhibited tumor growth in NC (group c) mice but showed limited efficacy in PLTP‐overexpressing (group d) tumors. Importantly, the combination of fenofibric acid and aPD‐1 significantly suppressed tumors in both the NC (group e) and PLTP (group f) groups, and there was no significant difference in tumor burden between the two groups at the end of the experiment, indicating that fenofibric acid reversed PLTP‐driven immunotherapy resistance. The subgroup analysis further supported these findings. In the NC group, aPD‐1 monotherapy led to noticeable tumor shrinkage, which was further enhanced by the addition of fenofibric acid, and some tumors completely regressed. In contrast, in the PLTP group, tumors in the combination therapy cohort were significantly smaller than those in the anti‐PD‐1 monotherapy group, highlighting the limited efficacy of PD‐1 blockade alone in PLTP‐overexpressing tumors and the potential of fenofibric acid to enhance ICIs responsiveness. Consistently, the survival analysis showed that combination therapy (groups e and f) significantly prolonged the overall survival of orthotopic HCC‐bearing mice compared to either anti‐PD‐1 (groups c and d) or IgG monotherapy (groups a and b) (Figure 6H). IHC analysis showed that compared to aPD‐1 treatment alone, fenofibric acid combined with aPD‐1 significantly reduced the infiltration of CD163+ and CD206+ macrophages (M2) and increased the infiltration of tumor‐infiltrating cytotoxic CD8+ T cells (Figure 6I).

These results indicated that fenofibrate can reverse the suppressive effects of PLTP in HCC immunotherapy. Overall, these findings suggest that fenofibrates have the potential to overcome the negative effects of PLTP on the effectiveness of aPD‐1 therapy. This indicates a promising avenue for improving the outcomes of immunotherapy for HCC treatment.

**Figure 7:**
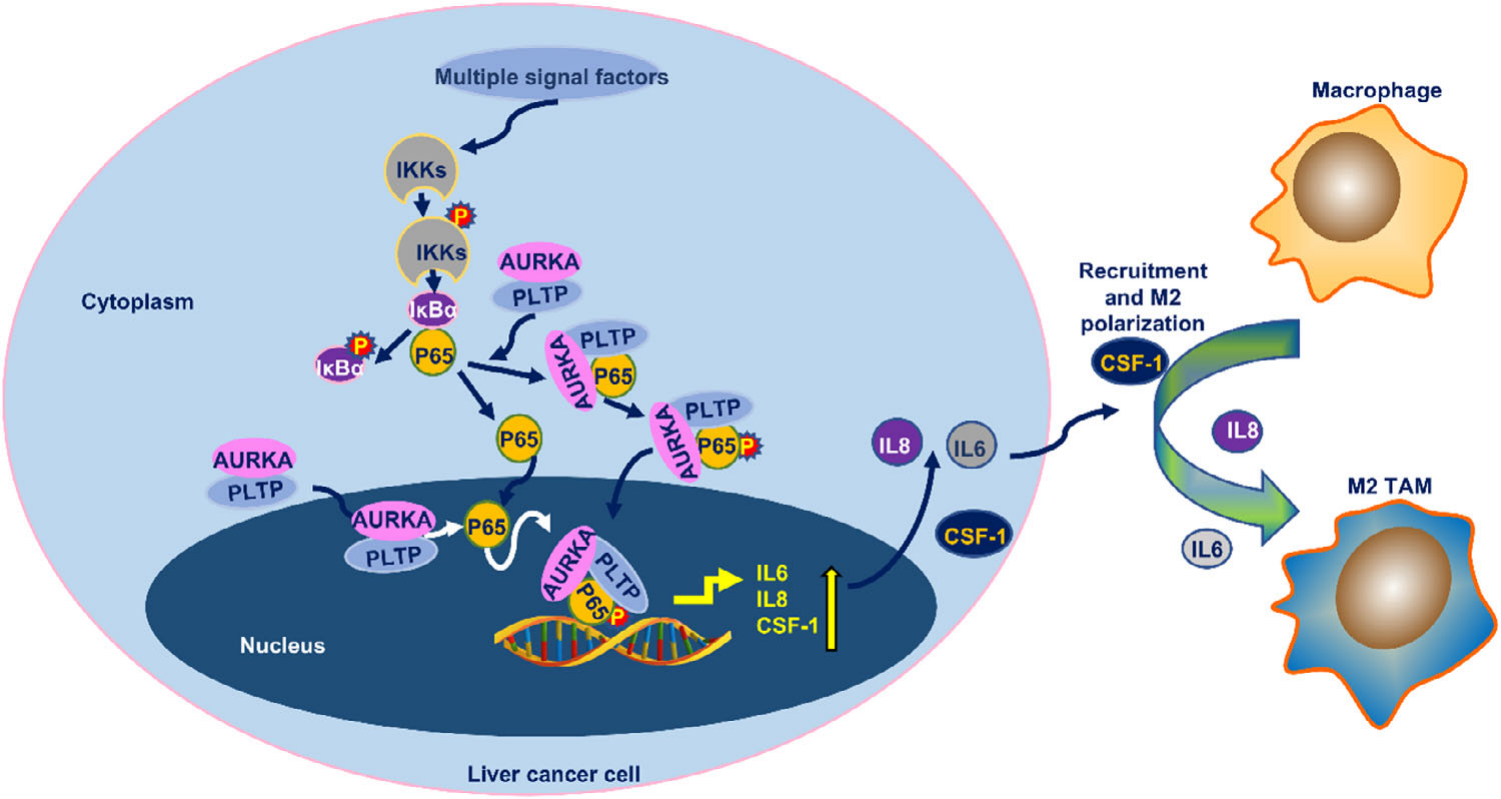
Schematic of the mechanism.

Our study demonstrates that PLTP plays a critical role in forming a complex with AURKA and P65. This interaction facilitates AURKA‐mediated phosphorylation of P65 at Ser536, leading to the activation of the NF‐κB signaling pathway. Ultimately, this leads to the upregulation of downstream cytokines, including IL‐6, IL‐8, and CSF‐1, which promotes M2 macrophage polarization and contributes to the immunosuppressive tumor microenvironment **Figure** **7**.

## 3 Discussion

Immunotherapy has significantly improved outcomes in patients with advanced HCC; however, its efficacy remains limited, and most initial responders eventually experience disease progression. Therefore, enhancing ICIs therapy and overcoming resistance are critical research priorities.^[^ ^33^ ^]^ The TME plays a crucial role in the immunotherapy response. HCC is shaped by chronic inflammation and is often linked to underlying liver diseases such as hepatitis B (HBV), hepatitis C (HCV), alcoholic cirrhosis, and metabolic‐associated fatty liver disease (MAFLD), fostering an immunosuppressive state.^[^ ^33^ ^]^ Dysregulated lipid metabolism is a hallmark of HCC and plays a pivotal role in tumor development and progression.^[^ ^34^ ^]^ Persistent lipid metabolic disturbances are closely linked to chronic inflammation, and these two processes reinforce one another.^[^ ^35^ ^]^ Many lipid metabolism molecules also serve as critical components of inflammatory pathways.

TAMs are the most prominent type of tumor‐infiltrating immune cells in the TME and play a critical role in the transition from early carcinogenesis to tumor progression.^[^ ^36^ ^]^ Macrophages exhibit high plasticity within the TME, ranging from pro‐inflammatory and anti‐tumor (M1‐like) to anti‐inflammatory and pro‐tumor (M2‐like) states. TAMs in HCC are predominantly skewed toward the M2 phenotype, which contributes to immune evasion and therapeutic resistance.^[^ ^37^ ^]^ Recent evidence has shown that M2 macrophages are critical drivers of resistance to ICIs, and patients with high levels of M2 macrophage infiltration are more likely to fail anti‐PD‐1 therapy.^[^ ^38^, ^39^ ^]^ Consequently, therapeutic strategies targeting M2 macrophages within the TME have emerged as promising novel approaches. In this study, we identified PLTP as a new biomarker that is negatively related to the prognosis of patients with HCC receiving ICIs treatment and elucidated a new mechanism by which PLTP mediates the phosphorylation of P65 through AURKA, thereby activating the NF‐κB signaling pathway. This activation promotes the infiltration and polarization of M2 macrophages in the HCC TME, resulting in tumor progression and limiting the efficacy of the anti‐PD‐1 therapy.

NF‐κB is a key mediator of inflammation and has been shown to regulate not only the expression of cytokines such as TNF‐α, IL‐6, IL‐1β, and COX‐2 but also lipid metabolism, including promoting adipose tissue inflammation and impairing cholesterol efflux. Collectively, these processes increase the risk of developing metabolic disorders. In cancer, NF‐κB signaling supports M2 macrophage polarization via the upregulation of IL‐10 and activation of STAT3, while also modulating glycolysis and fatty acid metabolism to sustain M2 macrophage function.^[^ ^40^ ^]^

PLTP, traditionally recognized as a lipid transporter, is found in various subcellular locations, including the cytoplasm, nucleus, extracellular matrix, and plasma.^[^ ^22^ ^]^ Our findings reveal a previously unrecognized role of PLTP in inflammation and immune modulation, independent of its lipid transfer activity. Through transcriptomic analysis and functional assays, we demonstrate that PLTP promotes NF‐κB‐mediated inflammation, macrophage recruitment, and M2 polarization. Interestingly, we showed that PLTP acts as a molecular adaptor that facilitates the formation of a PLTP‐P65‐AURKA complex, enabling AURKA to directly phosphorylate P65‐a novel finding supported by in vitro kinase assays. AURKA, a mitotic serine/threonine kinase, is often overexpressed in HCC and is associated with high‐grade tumors, p53 mutations, and poor prognosis.^[^ ^41^, ^42^ ^]^ AURKA is known to activate NF‐κB via phosphorylation of TIFA.^[^ ^43^, ^44^ ^]^ Our study is the first to demonstrate that AURKA directly phosphorylates P65, expanding our mechanistic understanding of this signaling axis.

Given the critical role of PLTP in mediating immune evasion and resistance to ICIs, we explored pharmacological strategies to inhibit its function. We first mapped the PLTP interaction domain with AURKA/P65 to amino acids 25‐245 and used molecular docking to screen for small molecules targeting this region. GMB‐475 is a proteolysis‐targeting chimera (PROTAC) designed to allosterically bind to the myristoyl pocket of ABL1, thereby inducing ubiquitin‐proteasome‐mediated degradation of BCR::ABL1. Previous studies demonstrated that GMB‐475, in combination with tyrosine kinase inhibitors (TKIs), synergistically inhibited cell proliferation and promoted apoptosis in chronic myeloid leukemia (CML) models harboring BCR::ABL1 mutations.^[^ ^45^, ^46^ ^]^ Further studies confirmed that GMB‐475 disrupted the PLTP‐P65‐AURKA complex and reversed PLTP‐driven tumor progression, highlighting the therapeutic potential of targeting this newly defined protein‐protein interaction axis.

To date, no drugs targeting the PLTP functional binding domain have been approved for human use. Considering that PLTP is a phospholipid transfer protein, we investigated whether clinically used antilipidemic drugs can effectively reduce PLTP, thereby inhibiting PLTP‐induced M2 macrophage infiltration and enhancing the efficacy of ICIs. Among the multiple antilipemic drugs available, fenofibrate effectively reduces endogenous PLTP expression and consequently inhibits the phosphorylation of P65. Consequently, fenofibrate inhibited M2 macrophage infiltration and enhanced PD‐1 efficiency in vivo of mice bearing HCC allografts (Figure 6). Our in vivo experimental results indicated the following aspects: (1) HCC tumors with high PLTP expression responded poorly to PD‐1 blockade alone, but this resistance was reversed by the co‐administration of fenofibrate, with survival curves aligned between the groups; and (2) even in tumors with low PLTP expression, fenofibrate synergized with PD‐1 blockade to prolong survival. Although our results confirmed that the mediating effect of PLTP interaction with P65 and AURKA is independent of its lipid metabolic activity, previous studies have demonstrated that fenofibrate suppresses PLTP expression at the transcriptional level,^[^ ^47^ ^]^ which in turn reduces total PLTP levels. Based on this, we speculated that fenofibrate may also exert its antitumor effects by interfering with lipid metabolism. Emerging evidence also supports the role of statins in cancer immunomodulation, potentially by inhibiting DNA replication and angiogenesis, or by modulating immune activity.^[^ ^48^, ^49^ ^]^ A key study by Yamasaki et al. demonstrated that fenofibrate significantly suppressed the growth of human HCC cells (Huh7 line) through a mechanism independent of PPARα activation.^[^ ^50^ ^]^ More recently, an important study further elucidated a novel antitumor mechanism of fenofibrate, showing that it suppresses hepatoma progression by downregulating the expression of osteopontin (OPN), a key tumor‐promoting cytokine, via inhibition of the PI3K/AKT/Twist signaling axis.^[^ ^51^ ^]^ These pleiotropic effects support the potential repurposing of lipid‐lowering agents to enhance the efficacy of immunotherapy, particularly in tumors with dysregulated lipid metabolism, such as HCC.

PLTP may play a multifaceted role in reshaping the HCC TME. Tumor cells and cancer stem cells secrete cytokines (e.g., IL‐6, IL‐8 and CSF‐1) and release extracellular vesicles carrying immunomodulatory molecules (e.g., M‐CSF, MIF and POSTN) that influence macrophage polarization.^[^ ^52^ ^]^ PLTP, with its ability to bind lipids and proteins, may act as a soluble mediator that traverses cell membranes and modulates stromal cell behavior. The novel function of PLTP as a secreted, bioactive factor warrants further investigation. This hypothesis warrants further investigation in future studies. Although PLTP is expressed in multiple tissues, the liver is the primary site of production,^[^ ^53^ ^]^ supporting the feasibility of targeting PLTP expression within liver tumors without significantly affecting systemic lipid metabolism. Tissue‐level assessment of PLTP may offer prognostic value and inform decisions regarding co‐treatment with immunotherapy and fenofibrate. The development of liver‐targeted drug delivery systems for PLTP inhibition may further refine this therapeutic strategy.

Nevertheless, the current study has several limitations that should be acknowledged: (1) The cohort analyzing the impact of PLTP on immunotherapy response consisted of only 55 patients, which represents a relatively small sample size; (2) the specific phosphorylation sites on P65 mediated by AURKA have not been fully elucidated, and additional sites may be involved; (3) Although GMB‐475 specifically binds to PLTP, its potential off‐target interaction with the ABL1 protein cannot be fully ruled out.

## 4 Conclusion

Our findings indicate that PLTP is a key regulator of the immunosuppressive microenvironment in HCC through the formation of a PLTP‐P65‐AURKA complex that activates NF‐κB signaling and promotes M2 macrophage polarization. This function is independent of PLTP's canonical lipid transfer activity. We provided the first evidence that P65 is a direct phosphorylation substrate of AURKA. The pharmacological targeting of PLTP using inhibitor GMB‐475 or fenofibrate reverses immune suppression and enhances ICIs efficacy. These findings offer mechanistic insights and a rationale for novel therapeutic strategies to overcome resistance to immunotherapy in HCC.

## 5 Experimental Section

### Dataset Information

RNA‐seq data and clinical information of TCGA‐LIHC patients were downloaded from UCSC XENA (http://xena.ucsc.edu/). Univariate Cox analysis was performed to identify poor prognostic‐related genes in HCC. The lists of immune‐related genes were obtained from the ImmPort database (https://www.immport.org/shared/home).

Univariate and multivariate Cox regression analysis and Kaplan–Meier analysis were used to evaluate the 22 immune cell infiltrations status of the TCGA‐LIHC cohort via The Tumor Immune Estimation Resource (TIMER2.0) website (http://timer.cistrome.org/).

Furthermore, Bladder Urothelial Carcinoma cohort Imvigor 210 (http://research‐pub.gene.com/IMvigor210CoreBiologies) was a cohort with complete clinical information and mRNA data and treated with anti‐PD1/PD‐L1 therapy. SMART‐seq2 data of single immune cells in the HCC map was used to explore the relationship between the immune cells and PLTP (http://cancer‐pku.cn:3838/HCC/).

### HCC Specimens

Formalin‐fixed, paraffin‐embedded (FFPE) tissue blocks were utilized in this study. These blocks were prepared from fresh tumor liver tissues collected from 55 patients with HCC who received immunotherapy and underwent hepatectomy at the Fifth Affiliated Hospital of Sun Yat‐Sen University between January 2018 and August 2024. All samples were independently examined and confirmed by two pathologists. This study was approved by the approval of the Medical Ethics Committee of The Fifth Affiliated Hospital of Sun Yat‐sen University (2024‐K281–1).

### Animals and Animal Models

SPF‐grade 4‐6‐week‐old female C57BL/6J mice were purchased from Zhuhai BaiShiTong Biotechnology. All animal experiments were approved by the Animal Protection and Use Committee of Sun Yat‐sen University (Guangzhou, China) and the Ethics Committee of the Fifth Affiliated Hospital of Sun Yat‐sen University.

A subcutaneous xenograft model was established by injecting 1 × 10^6 Hepa1‐6 cells in 200 µL PBS into the axillary region of mice. An orthotopic tumor model was created by injecting 2 × 10^6 HCC cells mixed with 20 µL of matrigel in PBS into the left lobe of the mouse liver. When the tumor volume in the experimental groups reached approximately 1.5 cm^3^, all mice were euthanized by CO_2_ inhalation. Tumors were excised, weighed, photographed, and the tumor formation in each group was recorded. Tumor tissues were fixed in 4% paraformaldehyde, paraffin‐embedded, sectioned, and later used for immunohistochemistry analysis.

Clodronate Liposomes and sterile PBS (for injection) were naturally warmed to room temperature, gently inverted and mixed. 200 µL of the mixture was injected intraperitoneally, and peripheral blood was collected 24 h later for flow cytometry to assess the macrophage depletion.

Alb‐Cre‐Tg and H11‐CAG‐LSL‐Myc mice were purchased from Shanghai Model Organisms Center, Inc. The H11‐LSL‐Myc mouse model was established by inserting the CAG promoter‐loxp‐STOP‐loxp‐Myc‐polyA conditional overexpression structure into the H11 locus, which was located on mouse chromosome 11 and can be used to express exogenous genes in a more extensive manner. MYC‐driven spontaneous HCC mice were generated by crossing the Alb‐Cre‐Tg with H11‐CAG‐LSL‐Myc mice at two months.

### Immunohistochemistry Staining (IHC)

Each tissue sections were deparaffinized, rehydrated, immersed in Tris‐EDTA antigen retrieval solution (Beyotime, Cat#P0084, 1:50 dilution) and heated at 100 °C for 10 min. They were then allowed to cool naturally to room temperature and washed three times with PBS for 5 min each. Next, the sections were incubated with hydrogen peroxide for 20 min, washed three times with PBS for 5 min each and blocked with 5% BSA at 37 °C for 30 min to block non‐specific binding, and the sections were incubated overnight at 4 °C with the following primary antibodies: PLTP (Abcam, Cat#ab282456, 1:500 dilution), CD163 (CST, Cat#93498S, 1:500 dilution), CD206 (CST, Cat#24 595, 1:500 dilution), CD8 (Abcam, Cat#ab245118, 1:1000 dilution). The next day, the sections were washed three times with PBS, the HRP conjugated mouse/rabbit IgG Super Vision Assay Kit (Boster, Cat#SV0001, SV0002) was incubated for an hour and then rewashed three times, DAB (Boster, Cat#AR1027) was added for 1–3 min to induce color development, the faucet rinsed to terminate, hematoxylin was counterstained for 3 min, dehydrated, cleared with xylene, covered with neutral resin, and finally observed under a microscope. The expression intensity score was 1 (weak), 2 (medium), 3 (strong), or 4 (very strong). The expression area score was also 1 (weak), 2 (medium), 3 (strong), or 4 (very strong). The comprehensive score was the product of the expression intensity score and the expression area score, ranging from 1 to 16. A score of 6 was considered the critical value. After evaluation by three inspectors, the majority consensus will determine the final score.

### Cell Culture

PLC/PRF/5, Hep3B, LM3, Hepa1‐6, THP‐1 cells derived from Procell. PLC/PRF/5, Hep3B, LM3, and Hepa1‐6 cells were maintained in Dulbecco's Modified Eagle's Medium (Gibco), supplemented with 10% (v/v) heat‐inactivated fetal bovine serum, 100 µg/mL of streptomycin, and 100 U/mL of penicillin (Hyclone). THP‐1 cells were cultured in RPMI 1640 medium (Gibco) supplemented with 10% (v/v) heat‐inactivated fetal bovine serum, 100 µg/mL of streptomycin, and 100 U/mL of penicillin. All cell lines were incubated at 37 °C in a humidified atmosphere containing 5% CO_2_.

### Differentiation of Macrophages

THP‐1 cells were induced to M0 macrophages by PMA (100 ng/mL) (MultiSciences) for 24 h at 37 °C. For M2 macrophages inducing, M0 macrophages were treated with IL‐4 (20 ng/mL) (PeproTech).^[^ ^54^, ^55^ ^]^ To observe the influence of HCC cells on M2 macrophages polarization, M0 macrophages were incubated with the culture supernatant of PLC/PRF/5 and Hep3B cells.

### Transfection

For transient overexpression, Liposomal Transfection Reagent (Yeasen) was applied according to the manufacturer's instructions to transfect cells in 60mm dishes containing plasmids or negative control. The cells and culture supernatant were harvested for subsequent experiments at 48 h posttransfection. To downregulate PLTP and AURKA, siRNA (Ribobio) was transiently transfected into PLC/PRF/5 or Hep3B cells. Briefly, when the cells reach 30‐50% confluence, scrambled siRNA (used as a negative control) or siRNA targeting PLTP or AURKA was added to the cell culture mixture using Lipomaster 2000 (Vazyme).

### Western Blot Analysis

Cells from different experimental groups were harvested and lysed in RIPA lysis buffer (RIPA 98%, EDTA 1%, and protease and phosphatase inhibitor 1%; CWBIO, Beyotime.), then centrifuged at 12,500 rpm for 15 min at 4 °C to collect the supernatant. The protein concentration was measured using a BCA protein assay kit (Beyotime) following the manufacturer's instructions. Then 5× loading buffer was added in and the protein was denatured at 100 °C for 6 min. Equal amounts of protein (20 µg) was separated by sodium dodecyl sulfate polyacrylamide gel electrophoresis on 10% gels and transferred to polyvinylidene difluoride membranes (0.45 µm; Merck Millipore). The blotting membrane was blocked in blocking solution (EpiZyme) for 30 min at room temperature, followed overnight incubation with the primary antibodies at 4 °C. After washing with PBST buffer, the membrane was then incubated with the secondary antibody (CST,1:10 000 dilution) for 1 h at room temperature. After an additional wash with PBST buffer, signals were developed using Western Lightning ECL Pro reagent (Vazyme). Blot signals were captured and quantified using a biomolecular imager.

### Transwell Migration Assay

The cell culture supernatant was filtered with a 0.45 µm filter (Jet) and 500 µL of supernatant was placed into the lower compartment of a 24‐well plate (Corning) equipped with an 8.0‐µm filter membrane, and 8 × 10^3 THP‐1 cells were seeded into the upper chamber in a humidified 5% CO_2_/95% air environment at 37 °C. After 24 h, the medium was discarded, the upper chamber was then fixed with paraformaldehyde for 20 min, stained with the crystal violet for 30 min, washed three times with PBS buffer followed by observation under the microscope.

### qRT‐PCR

Total RNA was extracted using the Total RNA Kit (Goonie), and cDNA was synthesized by reverse transcription using the Hifair III 1st Strand cDNA Synthesis SuperMix for qPCR (Yeasen) according to the manufacturer's instructions. qRT‐PCR was performed using the Applied Biosystems 7500 real‐time PCR system and the Hieff qPCR SYBR Green Master Mix (ROX) (Yeasen). The expression levels of all target genes were normalized to the expression of GAPDH, and fold changes were calculated using the 2^−ΔΔCt^ method. The primers used for qRT‐PCR were listed in Table S1 (Supporting Information).

### Flow Cytometry

Cells were digested with trypsin. For each group, 1 × 10^6 cells were harvested, washed twice with pre‐cooled PBS, and centrifuged at 1500 rpm for 3 min at 4 °C to collect the cells. The cells were fixed in 200 µL of fixation solution for 10 min at 4 °C. After two additional PBS washes, they were then resuspended in 50 µL of permeabilizer, and stained with the appropriate fluorochrome‐conjugated antibodies for 30 min at 4 °C in the dark. Afterward, they were washed twice with permeabilizer. Finally, the cells were resuspended in 300 µL of PBS, transferred to flow cytometry tubes, and analyzed using a flow cytometer.

### Immunofluorescence

The cells were fixed in 4% paraformaldehyde for 20 min at room temperature and washed three times with PBS. The cells were treated with 0.1% Triton X‐100 for 15 min at room temperature. The washed cells were incubated with a blocking serum for 1 h and then incubated overnight with primary antibody. The cells were then washed and incubated with secondary antibodies for 1 h in a dark room. For nuclear staining, the cells were incubated with 10 µL of DAPI for 10 min. The dishes were finally washed and mounted for microscopic examination.

### Co‐Immunoprecipitation (Co‐IP) Assay

Cells were harvested and lysed in 500 µL of Co‐IP lysis buffer containing a protease inhibitor mixture (Beyotime). After centrifugation, the cell lysates were collected and incubated overnight at 4 °C with the primary antibody or control immunoglobulin (Ig)G on a rotating mixer. The next day, 50 µL of immobilized Protein A/G beads (Beyotime) was added, and the protein complexes were precipitated at 4 °C for 4 h. The samples were then washed three times, and the beads were boiled in 1× loading buffer. The proteins were then prepared for Western blot analysis as described above.

### In Vitro Kinase Assay

Recombinant active AURKA protein (Abcam, Cat# ab271368‐10 µg) and recombinant human NF‐κB P65 protein (Abcam, Cat# ab114150‐10 µg) were used. The kinase reaction was carried out in kinase assay buffer (CST, Cat# 9802S) containing 200 µM ATP (Beyotime, Cat# D7378) at 37 °C for 30 min. The reaction was terminated by adding 5× loading buffer and boiling at 100 °C for 6 min. Phosphorylation of P65 was detected by Western blot using phospho‐specific antibodies against P65 (CST, Cat# 3033; phospho‐Ser536).

### Virtual Screening

Virtual screening was performed based on the predicted structure of human PLTP obtained from AlphaFold. A total of 10000 compounds from the TargetMol compound library (T001) and the Diversity Core Pre‐Plated Library (LF1000) were processed to generate small‐molecule conformers, followed by binding mode analysis. The virtual screening service was provided by TargetMol (Boston, MA, USA).

### Cellular Thermal Shift Assay (CETSA)

The CETSA was performed to evaluate drug‐target engagement in intact cells. Briefly, cells were collected, washed with cold PBS, and subjected to three cycles of freeze‐thaw lysis using liquid nitrogen to obtain total protein extracts. The lysates were then incubated with the GMB‐475 (MCE, Cat# 2490599‐18‐1) or DMSO control at room temperature for 1 h. After incubation, samples were centrifuged at 12000 × g for 20 min at 4 °C to remove insoluble material. The supernatant was aliquoted into PCR tubes and subjected to a temperature gradient (from 37 °C to 67 °C) for 3 min, followed by cooling at room temperature for 3 min. Subsequently, the heated samples were centrifuged at 20000 × g for 30 min at 4 °C to separate aggregated proteins. The supernatant was collected and mixed with 5× loading buffer, denatured at 100 °C for 6 min, and then analyzed by Western blot to detect the stability of the target protein upon ligand binding.

### GST Pull‐Down Assay

Recombinant GST protein (GST) and His‐tagged binding partners (His‐P65) were expressed in *E. coli* Rosetta2(DE3) (Beyotime, Cat#D1067S) and purified using GST‐tag Protein Purification Kit (Beyotime, Cat#P2262) or His‐tag Protein Purification Kit (Beyotime, Cat#P2226), respectively. Recombinant Human Phospholipid transfer protein (PLTP) (CSB‐EP018212HU) and recombinant Human Aurora kinase A (AURKA) (CSB‐EP002454HU) were purchased from Cusabio. Approximately 10 µg of purified GST or GST‐PLTP protein bound to glutathione beads was incubated with 10 µg of the candidate interacting protein in 500 µL of binding buffer for 2 h at 4 °C with gentle rotation. Beads were then washed five times with ice‐cold binding buffer to remove non‐specifically bound proteins. Bound proteins were eluted by boiling in 2× loading buffer for 10 min and analyzed by SDS‐PAGE and immersed in Coomassie Brilliant Blue staining solution for 1 h with gentle shaking.

### Statistical Analysis

All experiments were independently repeated three times. Statistical analysis was carried out using R (version 4.2.0, https://www.r‐proje ct.org/) or GraphPad Prism 9.0.0 (GraphPad Software Inc., San Diego, CA, USA). Categorical data were analyzed, and comparisons of continuous variables were performed with the chi‐squared/Fisher's exact test and Student's *t*‐test/Mann–Whitney *U* test, respectively. *P* < 0.05 was considered statistically significant. Before the survival analysis, the mean expression level was used to classify patient samples into two groups. Kaplan–Meier survival analysis was performed to assess the associations of gene expression levels with clinical outcomes, and *P*‐values were calculated with the log‐rank test.

## 6 Ethics Declarations

Ethical approval was granted by the Fifth Affiliated Hospital of Sun Yat‐Sen University Ethics Committee, and all procedures adhered to the International Ethical Guidelines for Biomedical Research Involving Human Subjects (CIOMS) (2024‐K281–1). Ethical approval for all animal procedures was granted by the Institutional Animal Care and Use Committee of the Fifth Affiliated Hospital of Sun Yat‐Sen University (00420).

## Author Contributions

X.L., Y.L., J.C., and L.L. contributed equally to this work. X.L., Y.L., J.C. and L.L. performed conceptualization. P.Y., Y.C., J.Z., Y.Z., J.Y., W.Z., Y.L. performed methodology. X.L., Y.L., J.C. and L.L. performed investigation and visualization. W.P., C.W., L.Z. performed funding acquisition. C.W., J.L., L.Z. performed supervision. X.L., Y.L., J.C. and L.L. performed writing & editing.

## Conflict of Interest

The authors declare no conflict of interests.

## Supporting information



Supporting Information

## Data Availability

The data that support the findings of this study are available from the corresponding author upon reasonable request.

## References

[1] J. M. Llovet , R. K. Kelley , A. Villanueva , A. G. Singal , E. Pikarsky , S. Roayaie , R. Lencioni , K. Koike , J. Zucman‐Rossi , R. S. Finn , Nat. Rev. Dis. Primers 2021, 7, 6. 33479224 10.1038/s41572-020-00240-3 PMC13537433

[2] C. Yang , H. Zhang , L. Zhang , A. X. Zhu , R. Bernards , W. Qin , C. Wang , Nat. Rev. Gastroenterol. Hepatol. 2023, 20, 203. 36369487 10.1038/s41575-022-00704-9

[3] E. J. Wherry , M. Kurachi , Nat. Rev. Immunol. 2015, 15, 486. 26205583 10.1038/nri3862 PMC4889009

[4] S. C. Wei , C. R. Duffy , J. P. Allison , Cancer Discov. 2018, 8, 1069. 30115704 10.1158/2159-8290.CD-18-0367

[5] S. L. Topalian , C. G. Drake , D. M. Pardoll , Curr. Opin. Immunol. 2012, 24, 207. 22236695 10.1016/j.coi.2011.12.009 PMC3319479

[6] S. Halle , O. Halle , R. Förster , Trends Immunol. 2017, 38, 432. 28499492 10.1016/j.it.2017.04.002

[7] J. M. Llovet , S. Ricci , V. Mazzaferro , P. Hilgard , E. Gane , J.‐F. Blanc , A. C. de Oliveira , A. Santoro , J.‐L. Raoul , A. Forner , M. Schwartz , C. Porta , S. Zeuzem , L. Bolondi , T. F. Greten , P. R. Galle , J.‐F. Seitz , I. Borbath , D. Häussinger , T. Giannaris , M. Shan , M. Moscovici , D. Voliotis , J. Bruix , N. Engl. J. Med. 2008, 359, 378. 18650514 10.1056/NEJMoa0708857

[8] M. Kudo , R. S. Finn , S. Qin , K.‐H. Han , K. Ikeda , F. Piscaglia , A. Baron , J.‐W. Park , G. Han , J. Jassem , J. F. Blanc , A. Vogel , D. Komov , T. R. J. Evans , C. Lopez , C. Dutcus , M. Guo , K. Saito , S. Kraljevic , T. Tamai , M. Ren , A.‐L. Cheng , Lancet 2018, 391, 1163. 29433850 10.1016/S0140-6736(18)30207-1

[9] S. K. Biswas , A. Mantovani , Nat. Immunol. 2010, 11, 889. 20856220 10.1038/ni.1937

[10] G. Erdag , J. T. Schaefer , M. E. Smolkin , D. H. Deacon , S. M. Shea , L. T. Dengel , J. W. Patterson , C. L. Slingluff , Cancer Res. 2012, 72, 1070. 22266112 10.1158/0008-5472.CAN-11-3218 PMC3306813

[11] D. F. Quail , J. A. Joyce , Clin. Cancer Res. 2017, 23, 876. 27895033 10.1158/1078-0432.CCR-16-0133 PMC5453188

[12] J. M. Brown , L. Recht , S. Strober , Clin. Cancer Res. 2017, 23, 3241. 28341752 10.1158/1078-0432.CCR-16-3122 PMC5529121

[13] T. Beltraminelli , M. De Palma , J. Pathol. 2020, 250, 573. 32086811 10.1002/path.5403

[14] C. E. Lewis , J. W. Pollard , Cancer Res. 2006, 66, 605. 16423985 10.1158/0008-5472.CAN-05-4005

[15] A. J. Boutilier , S. F. Elsawa , Int. J. Mol. Sci. 2021, 22, 6995. 34209703 10.3390/ijms22136995 PMC8268869

[16] Q. He , M. Liu , W. Huang , X. Chen , B. Zhang , T. Zhang , Y. Wang , D. Liu , M. Xie , X. Ji , M. Sun , D. Tian , L. Xia , Hepatology 2021, 74, 3174. 34288020 10.1002/hep.32062

[17] S. Wan , E. Zhao , I. Kryczek , L. Vatan , A. Sadovskaya , G. Ludema , D. M. Simeone , W. Zou , T. H. Welling , Gastroenterology 2014, 147, 1393. 25181692 10.1053/j.gastro.2014.08.039 PMC4253315

[18] K. Pang , Z.‐D. Shi , L.‐Y. Wei , Y. Dong , Y.‐Y. Ma , W. Wang , G.‐Y. Wang , M.‐Y. Cao , J.‐J. Dong , Y.‐A. Chen , P. Zhang , L. Hao , H. Xu , D. Pan , Z.‐S. Chen , C.‐H. Han , Drug Resist Updat. 2023, 66, 100907. 36527888 10.1016/j.drup.2022.100907

[19] E. Peranzoni , J. Lemoine , L. Vimeux , V. Feuillet , S. Barrin , C. Kantari‐Mimoun , N. Bercovici , M. Guérin , J. Biton , H. Ouakrim , F. Régnier , A. Lupo , M. Alifano , D. Damotte , E. Donnadieu , Proc. Natl. Acad. Sci. USA 2018, 115, E4041. 29632196 10.1073/pnas.1720948115 PMC5924916

[20] Q. Han , H. Shi , F. Liu , Int. Immunopharmacol. 2016, 34, 101. 26938675 10.1016/j.intimp.2016.01.023

[21] L. J. Beamer , S. F. Carroll , D. Eisenberg , Biochem. Pharmacol. 1999, 57, 225. 9890549 10.1016/s0006-2952(98)00279-2

[22] T. Gautier , V. Deckert , M. Nguyen , C. Desrumaux , D. Masson , L. Lagrost , Pharmacol. Ther. 2022, 236, 108105. 34974028 10.1016/j.pharmthera.2021.108105

[23] C. Desrumaux , S. Lemaire‐Ewing , N. Ogier , A. Yessoufou , A. Hammann , A. Sequeira‐Le Grand , V. Deckert , J.‐P. Pais de Barros , N. Le Guern , J. Guy , N. A. Khan , L. Lagrost , Cell Mol. Immunol. 2016, 13, 795. 26320740 10.1038/cmi.2015.75 PMC5101448

[24] K. Zhang , X. Liu , Y. Yu , T. Luo , L. Wang , C. Ge , X. Liu , J. Song , X. Jiang , Y. Zhang , S. Qin , M. Zhang , Arterioscler Thromb Vasc. Biol. 2014, 34, 2537. 25324570 10.1161/ATVBAHA.114.303966

[25] D. Yan , M. Navab , C. Bruce , A. M. Fogelman , X.‐C. Jiang , J. Lipid Res. 2004, 45, 1852. 15258196 10.1194/jlr.M400053-JLR200

[26] J. Andreasson , E. Bodén , M. Fakhro , C. von Wachter , F. Olm , M. Malmsjö , O. Hallgren , S. Lindstedt , Respir. Res. 2022, 23, 369. 36544145 10.1186/s12931-022-02302-4 PMC9768396

[27] Q. Zhu , Y. Chai , L. Jin , Y. Ma , H. Lu , Y. Chen , W. Feng , Sci. Rep. 2023, 13, 18226. 37880277 10.1038/s41598-023-45289-8 PMC10600204

[28] H. Ke , R. Yuan , H. Liu , M. Luo , H. Hu , E. Zhang , K.e Zhuang , Y. Yang , R. Yang , Front. Immunol. 2023, 14, 1282469. 38022651 10.3389/fimmu.2023.1282469 PMC10667720

[29] K. Huang , S. Chen , R. Xie , P. Jiang , C. Yu , J. Fang , X. Liu , F. Yu , FEBS Open Bio. 2020, 10, 1891. 10.1002/2211-5463.12943 PMC7459413 32735728

[30] Y. Z. Chen , J. Y. Xue , C. M. Chen , B. L. Yang , Q. H. Xu , F. Wu , F. Liu , X. Ye , X. Meng , G. Y. Liu , Z. Z. Shen , Z. M. Shao , J. Wu , Cancer Chemother. Pharmacol. 2012, 70, 637. 22903535 10.1007/s00280-012-1949-0

[31] H. Sakurai , S. Suzuki , N. Kawasaki , H. Nakano , T. Okazaki , A. Chino , T. Doi , I. Saiki , J. Biol. Chem. 2003, 278, 36916. 12842894 10.1074/jbc.M301598200

[32] J. F. Oram , G. Wolfbauer , C. Tang , W. S. Davidson , J. J. Albers , J. Biol. Chem. 2008, 283, 11541. 18287097 10.1074/jbc.M800117200 PMC2431062

[33] K.‐Y. Shen , Y. Zhu , S.‐Z. Xie , L.‐X. Qin , J. Hematol. Oncol. 2024, 17, 25. 38679698 10.1186/s13045-024-01549-2 PMC11057182

[34] B. Paul , M. Lewinska , J. B. Andersen , JHEP Rep. 2022, 4, 100479. 35469167 10.1016/j.jhepr.2022.100479 PMC9034302

[35] H. Fu , B. Tang , J. Lang , Y. Du , B. Cao , L. Jin , M. Fang , Z. Hu , C. Cheng , X. Liu , Q. Shou , Front. Nutr. 2020, 7, 585306. 33304918 10.3389/fnut.2020.585306 PMC7701255

[36] M. Locati , G. Curtale , A. Mantovani , Annu. Rev. Pathol. 2020, 15, 123. 31530089 10.1146/annurev-pathmechdis-012418-012718 PMC7176483

[37] K. Cheng , N. Cai , J. Zhu , X. Yang , H. Liang , W. Zhang , Cancer Commun. 2022, 42, 1112. 10.1002/cac2.12345 PMC9648394 36069342

[38] D. G. DeNardo , B. Ruffell , Nat. Rev. Immunol. 2019, 19, 369. 30718830 10.1038/s41577-019-0127-6 PMC7339861

[39] Y. Zhu , J. Yang , D. Xu , X.‐M. Gao , Z. Zhang , J. L. Hsu , C.‐W. Li , S.‐O. Lim , Y.‐Y. Sheng , Y. Zhang , J.‐H. Li , Q. Luo , Y. Zheng , Y. Zhao , L. Lu , H.‐L. Jia , M.‐C. Hung , Q.‐Z. Dong , L.‐X. Qin , Gut 2019, 68, 1653. 30902885 10.1136/gutjnl-2019-318419

[40] A. Mantovani , P. Allavena , A. Sica , F. Balkwill , Nature 2008, 454, 436. 18650914 10.1038/nature07205

[41] R. Du , C. Huang , K. Liu , X. Li , Z. Dong , Mol. Cancer 2021, 20, 15. 33451333 10.1186/s12943-020-01305-3 PMC7809767

[42] Y.‐M. Jeng , S.‐Y. Peng , C.‐Y. Lin , H.‐C. Hsu , Clin. Cancer Res. 2004, 10, 2065. 15041727 10.1158/1078-0432.ccr-1057-03

[43] T.‐Y. W. Wei , P.‐Y. Wu , T.‐J. Wu , H.‐A. Hou , W.‐C. Chou , C.‐L. J. Teng , C.‐R. Lin , J.‐M. M. Chen , T.‐Y. Lin , H.‐C. Su , C.‐C. F. Huang , C.‐T. R. Yu , S.‐L. Hsu , H.‐F. Tien , M.‐D. Tsai , Cancer Res. 2017, 77, 494. 28069801 10.1158/0008-5472.CAN-16-1004

[44] F. D. Naso , D. Boi , C. Ascanelli , G. Pamfil , C. Lindon , A. Paiardini , G. Guarguaglini , Oncogene 2021, 40, 3917. 33981003 10.1038/s41388-021-01766-w PMC8195736

[45] W. Ye , X. Wu , X. Wang , X. Wei , Y. Tang , X. Ouyang , Y. Gong , Front. Pharmacol. 2022, 13, 931772. 36263131 10.3389/fphar.2022.931772 PMC9574342

[46] G. M. Burslem , A. R. Schultz , D. P. Bondeson , C. A. Eide , S. L. Savage Stevens , B. J. Druker , C. M. Crews , Cancer Res. 2019, 79, 4744. 31311809 10.1158/0008-5472.CAN-19-1236 PMC6893872

[47] A. Y. Tu , J. J. Albers , Biochem. Biophys. Res. Commun. 1999, 264, 802. 10544012 10.1006/bbrc.1999.1597

[48] M. O. Murto , N. Simolin , O. Arponen , A. Siltari , M. Artama , K. Visvanathan , A. Jukkola , T. J. Murtola , JAMA Netw. Open 2023, 6, 2343861. 10.1001/jamanetworkopen.2023.43861 PMC10656638 37976058

[49] B. Relja , F. Meder , M. Wang , R. Blaheta , D. Henrich , I. Marzi , M. Lehnert , Int. J. Oncol. 2011, 38, 879. 21206971 10.3892/ijo.2010.892

[50] D. Yamasaki , N. Kawabe , H. Nakamura , K. Tachibana , K. Ishimoto , T. Tanaka , H. Aburatani , J. Sakai , T. Hamakubo , T. Kodama , T. Doi , Eur. J. Cell Biol. 2011, 90, 657. 21514001 10.1016/j.ejcb.2011.02.005

[51] W. Chen , F. Chen , M. Gong , L. Ye , D. Weng , Z. Jin , J. Wang , Naunyn. Schmiedebergs Arch. Pharmacol. 2024, 397, 1025. 37566308 10.1007/s00210-023-02604-4 PMC10791796

[52] D. Li , L. Wang , B. Jiang , Y. Jing , X. Li , Biomed. Pharmacother. 2024, 170, 116043. 38128186 10.1016/j.biopha.2023.116043

[53] A. Yazdanyar , X. C. Jiang , Hepatology 2012, 56, 576. 22367708 10.1002/hep.25648 PMC3409695

[54] C. Zhu , D. Mustafa , P. P. Zheng , M. van der Weiden , A. Sacchetti , M. Brandt , I. Chrifi , D. Tempel , P. J. M. Leenen , D. J. Duncker , C. Cheng , J. M. Kros , Neurol. Oncol. 2017, 19, 648. 10.1093/neuonc/now251 PMC5464467 28453746

[55] G. S. Schulert , N. Fall , J. B. Harley , N. Shen , D. J. Lovell , S. Thornton , A. A. Grom , Arthritis Rheumatol. 2016, 68, 2300. 27014994 10.1002/art.39694 PMC5001902

