# Supplementary material for: Fibrates Inhibit PLTP‐induced M2 Macrophage Infiltration and Increase the Sensitivity of Hepatocellular Carcinoma to ICIs
Source: Adv Sci (Weinh). 2025 Dec 14;13(35):e13257. doi: 10.1002/advs.202513257 (PMC13292255; doi:10.1002/advs.202513257)
Supplement: Supplementary file 1 — Supporting Information [file ADVS-13-e13257-s001.docx]

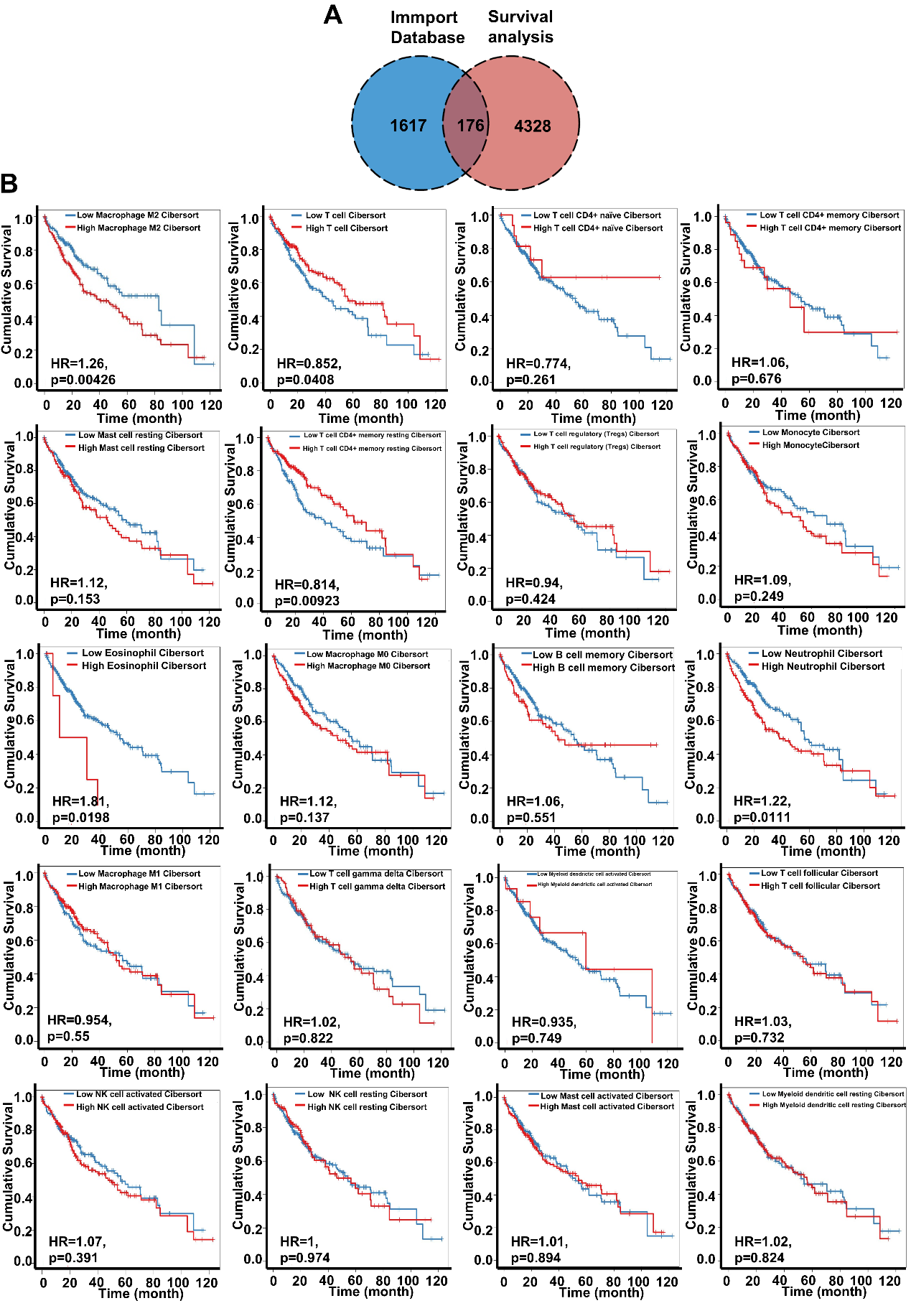


Figure S1. (A) Immune genes correlated with poor outcome genes screened out by Venn diagrams. (B) Kaplan-Meier analysis showing the relationship between prognosis and tumor-infiltrating immune cells by the CIBERSORT algorithm.


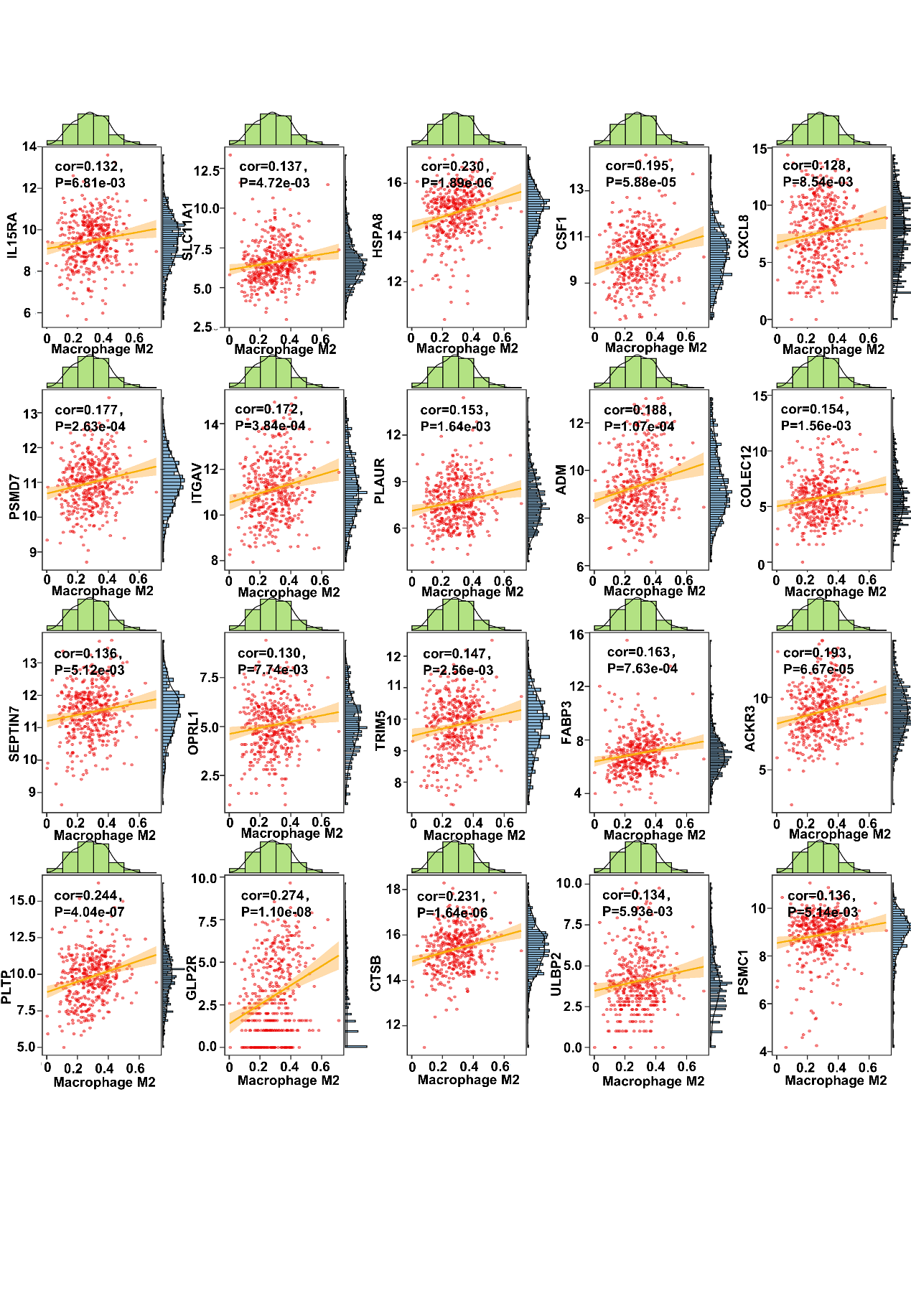


Figure S2. The correlation between M2 macrophage Infiltration and immune genes correlated to poor prognosis by Timer database.


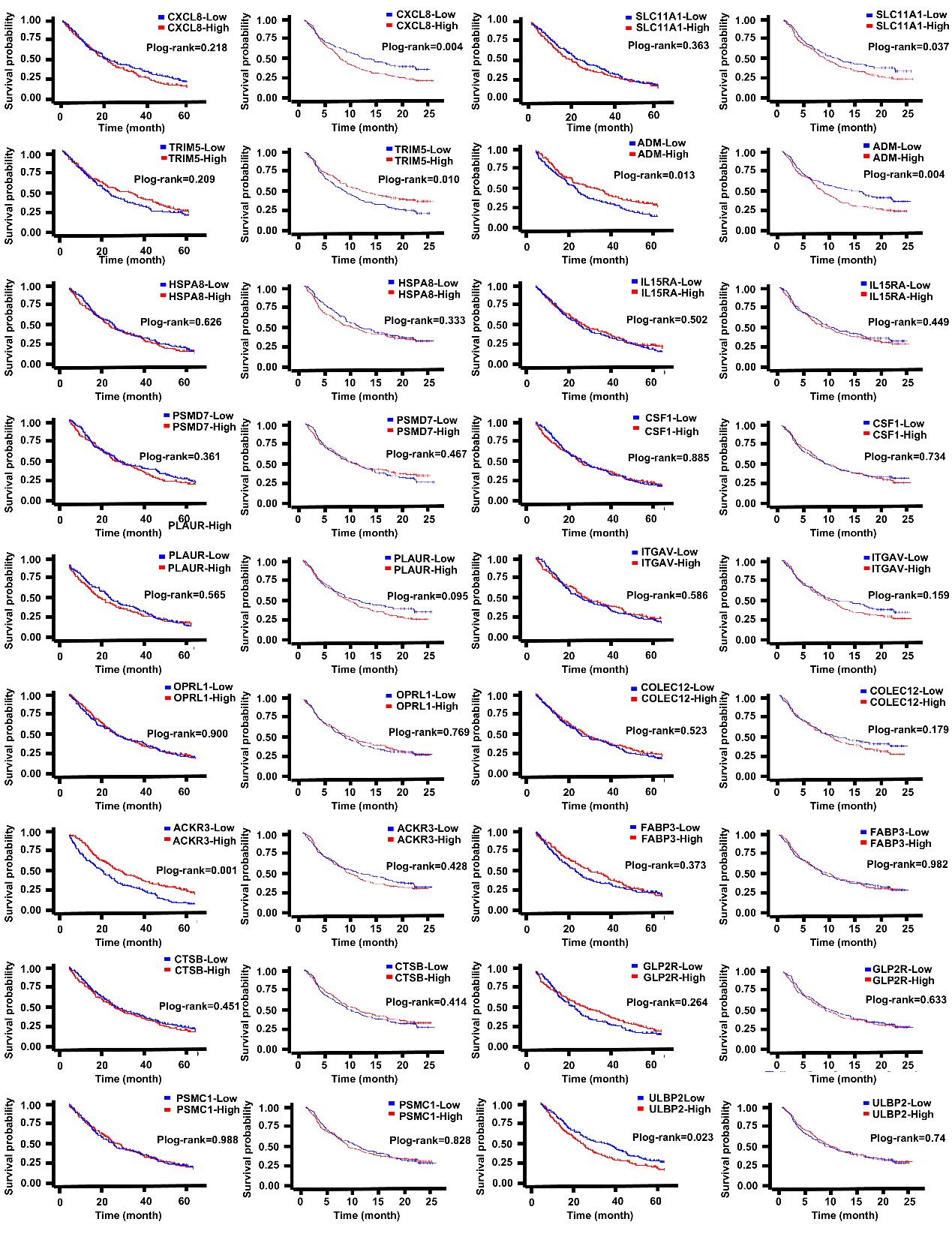


Figure S3. Kaplan−Meier plot for the expression of 20 immune genes and OS in the checkmate025 cohort (left) and Imvigor 210 cohort (right). (SEPTIN7 cannot be found).


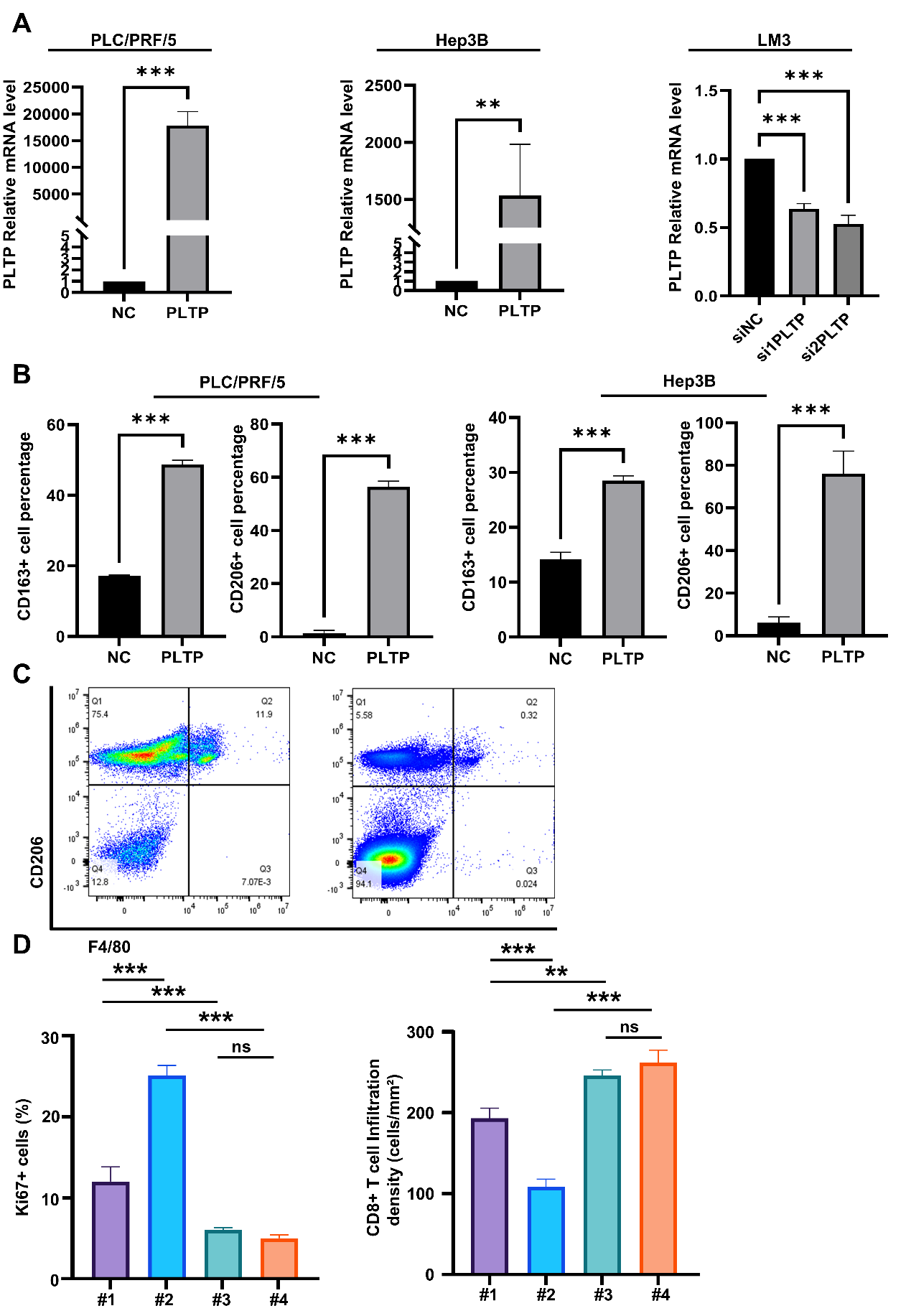


Figure S4. (A) qRT-PCR analysis of changes in mRNA levels of PLTP in multiple human HCC cell lines after transfection of PLTP-overexpressed plasmid or PLTP-knockdown siRNAs. (B) Flow cytometric analysis showed a significant increase in the expression of M2 markers (CD163 and CD206) in macrophages co-cultured with PLTP-overexpressing HCC-CM. (C) Flow cytometry analysis of macrophage depletion in peripheral blood of mice. (D) Quantification of the percentage of Ki67+ cells and the infiltration density of CD8+ cells by IHC in subcutaneous tumor tissues from C57BL/6J mice. **P* < 0.05, ***P* < 0.01, ****P* < 0.001.


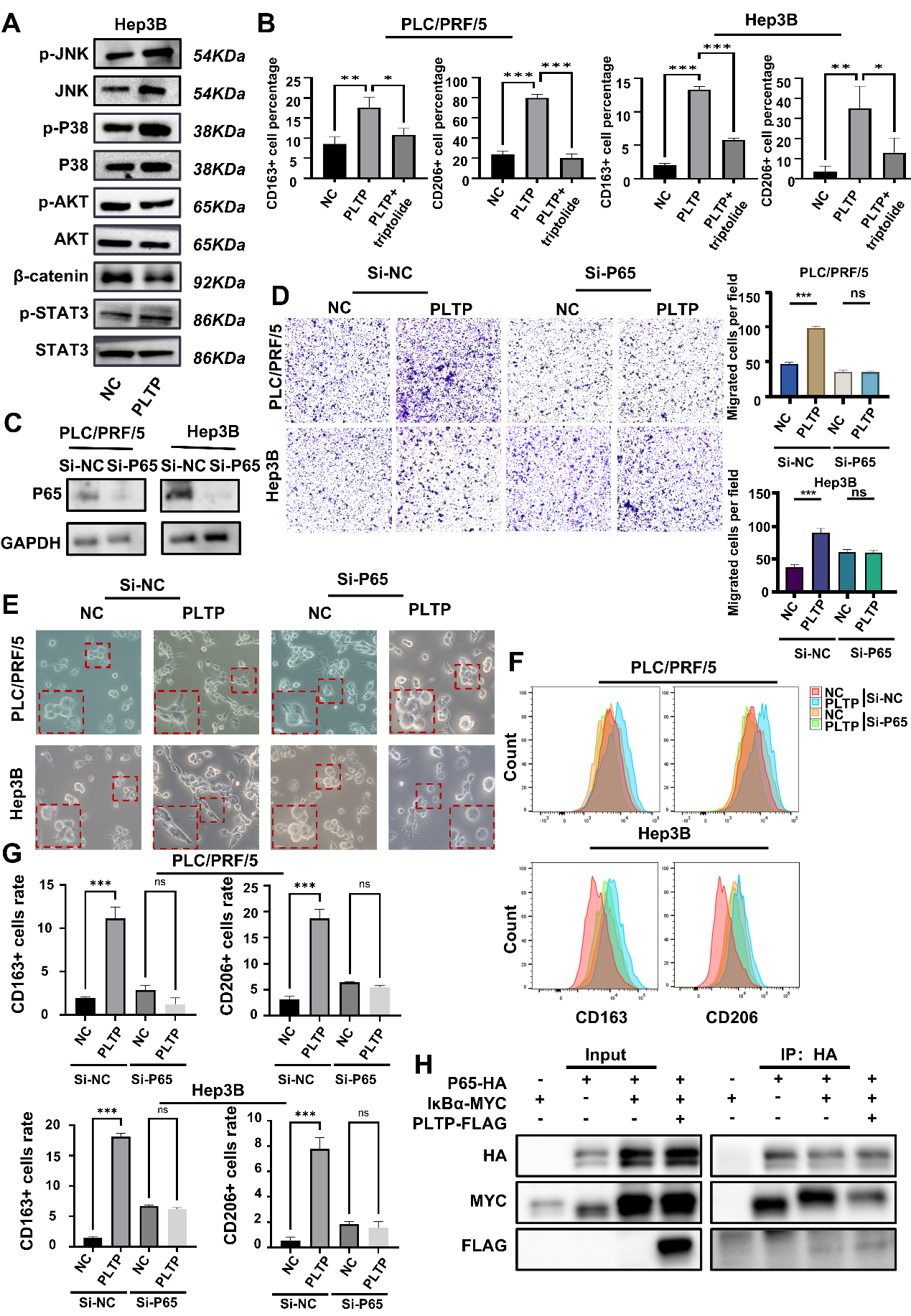


Figure S5. (A) Western blot analysis of phosphorylated JNK, P38, AKT, STAT3, and total β-catenin levels in Hep3B cells following PLTP overexpression. (B) Flow cytometry shows that PLTP-overexpressing HCC-CM increases CD163+ and CD206+ macrophages, an effect reversed by triptolide treatment. (C) P65 protein expression in HCC cell lines following transfection with P65-targeting siRNAs. (D) Transwell migration assay showing that PLTP-induced recruitment of THP-1 cells is attenuated upon P65 knockdown. (E) Microscopic analysis reveals that P65 knockdown inhibits the morphological polarization of M0 macrophages toward the M2 phenotype induced by PLTP-HCC CM. (F, G) Flow cytometry analysis shows increased numbers of CD163+ and CD206+ macrophages after co-culture with PLTP-HCC CM, whereas P65 knockdown inhibits the polarization of M0 macrophages toward the M2 phenotype induced by PLTP-HCC CM. (H) Exogenous competitive Co-IP experiment shows that increased PLTP expression led to a reduction in P65-IκBα binding. **P* < 0.05, ***P* < 0.01, ****P* < 0.001.


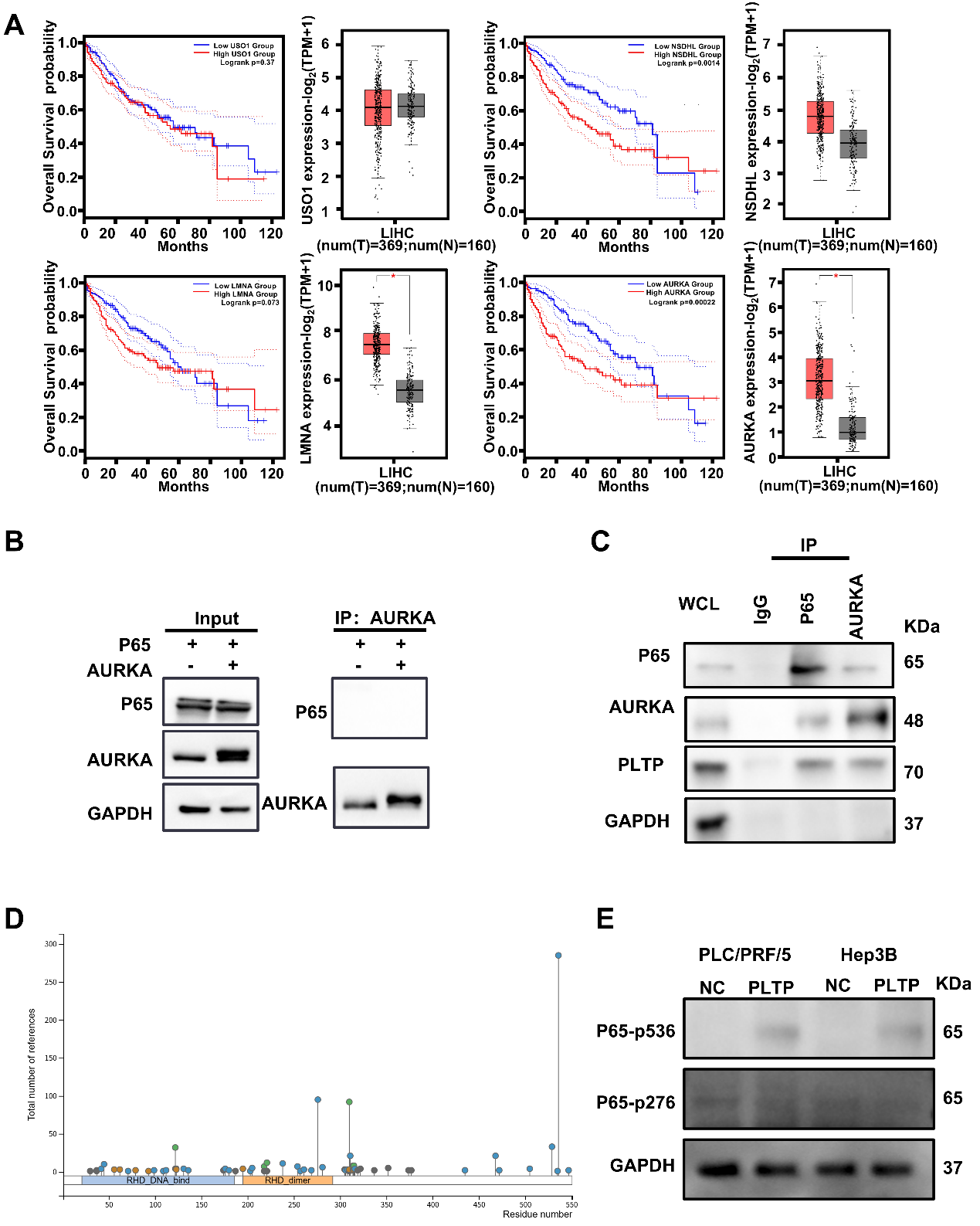


Figure S6. (A) Kaplan–Meier survival curves from GEPIA database showing the OS of LIHC patients stratified by high (red) and low (blue) expression levels of USO1, NSDHL, LMNA, and AURKA. The log-rank p-values are indicated; Box plots comparing mRNA expression levels (log2 TPM+1) of USO1, NSDHL, LMNA, and AURKA between tumor tissues (T, red) and normal tissues (N, gray) in the LIHC cohort from TCGA (tumor: n = 369; normal: n = 50). (B) Co-IP experiment demonstrates that P65 couldn’t bind to AURKA without PLTP. (C) Endogenous Co-IP assays demonstrate the interaction among PLTP, AURKA and P65. (D) PhosphoSitePlus® database shows P65-ser276 and ser536 are among the most extensively studied. (E) Western blot analysis of phosphorylated P65-ser276 and ser536 in HCC cells following PLTP overexpression.


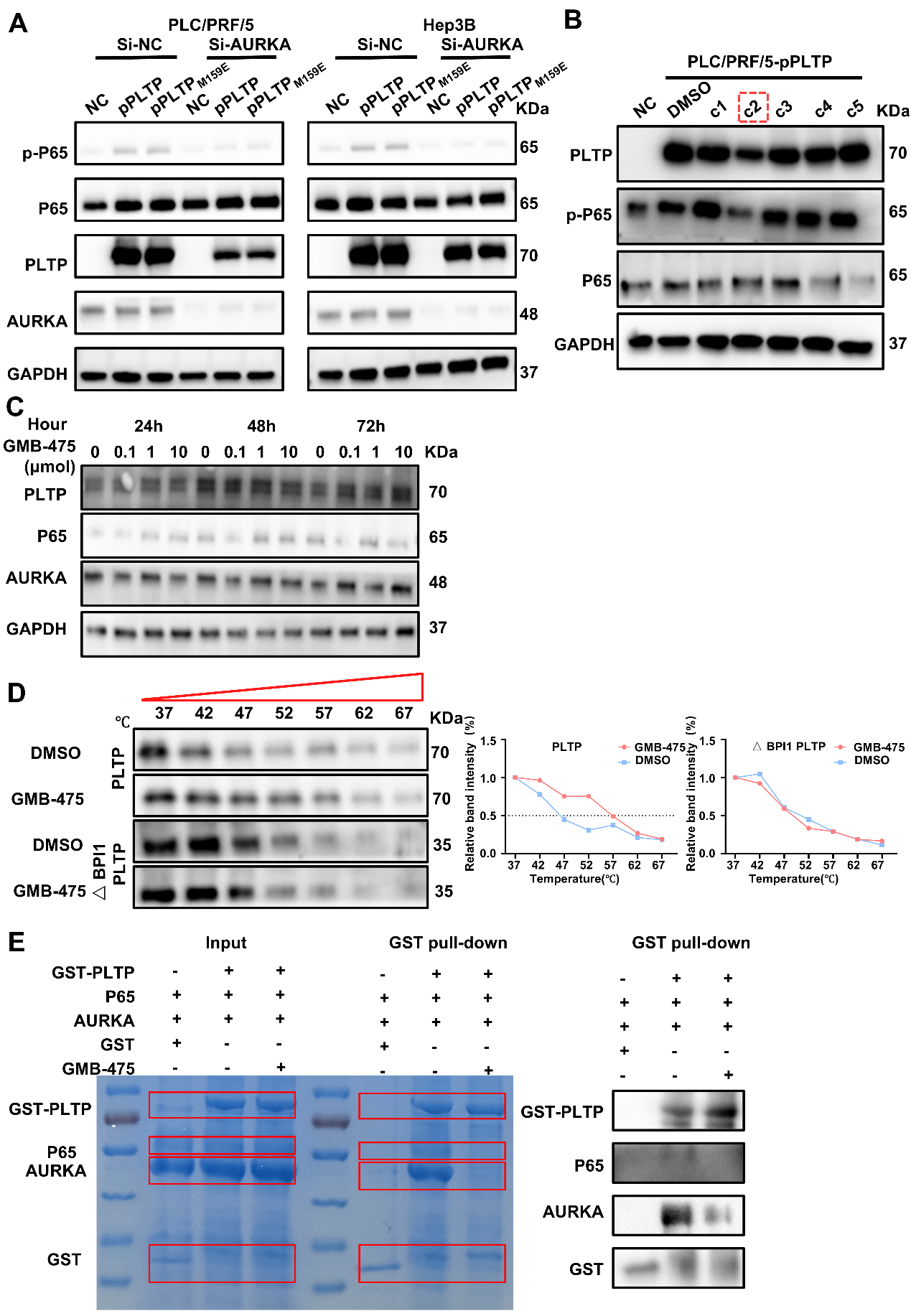


Figure S7. (A) Western blot analysis shows that AURKA knockdown suppresses PLTP and PLTP-M159E induced P65 phosphorylation in HCC cells. (B) Western blot analysis of p-P65 levels in PLC/PRF/5 cells overexpressing PLTP and treated with the top five PLTP-binding compounds (C1–C5) ranked by binding affinity. (C) Western blot analysis demonstrates GMB-475 does not induce degradation of PLTP, AURKA and P65 in a time- or dose-dependent manner. (D) Cellular Thermal Shift Assay (CETSA) reveals that GMB-475 stabilizes PLTP compared to the DMSO control, but does not stabilize the △BPI1 mutant form. (E) Pull-down assays showed that GMB-475 disrupted the PLTP-P65-AURKA interactions.


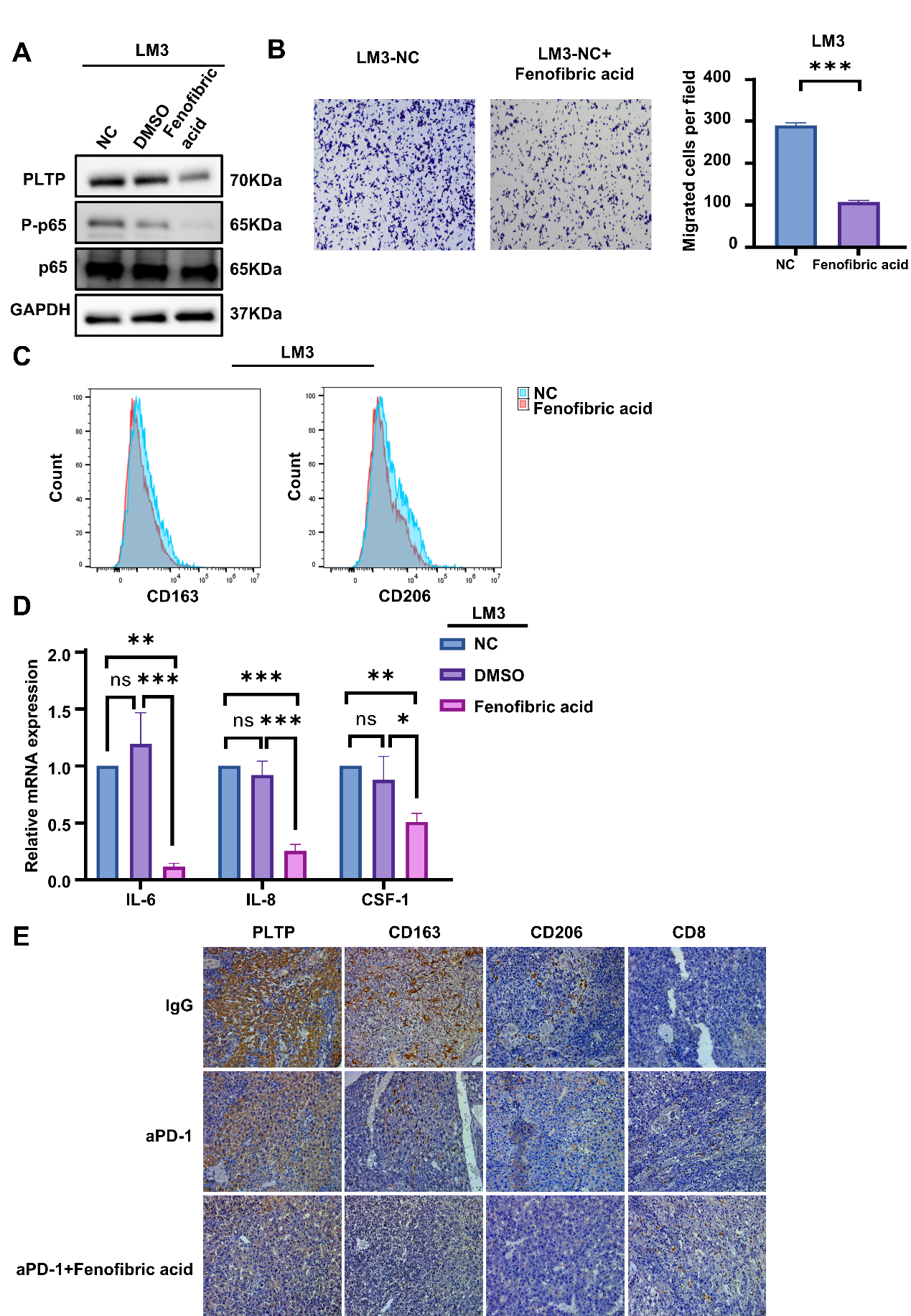


Figure S8. (A) Western blot analysis was performed to assess the expression levels of p-P65 in LM3 cells following treatment with fenofibric acid. (B) Fenofibric acid inhibits LM3 cell recruitment of THP-1 cells. (C) Fenofibric acid inhibits the polarization of M2 macrophages induced by LM3 cells. (D) qRT-PCR was performed to evaluate the changes in mRNA expression levels of IL-6, IL-8, and CSF-1 in LM3 cells following treatment with fenofibric acid. (E) Representative IHC images of PLTP, CD163, and CD8 expression in tumor harvested from Alb-Cre⁺/MYC⁺ mice with IgG, aPD-1, and aPD-1 + fenofibric acid therapy. Scale bar: 200 μm (magnification ×200).

| Name | Sequence（5‘-3’） |
| --- | --- |
| PLTP forward | CTCTCCACGTTCATCACCTCA |
| PLTP reverse | AATGCCAACAAGCTCGTCCA |
| Beta-actin forward | GGTCATCACTATTGGCAACG |
| Beta-actin reverse | ACGGATGTCAACGTCACACT |
| CD163 forward | CATGTCTCTGAGGCTGACCA |
| CD163 reverse | TGCACACGATCTACCCACAT |
| CD206 forward | TACCTGAGCCCACACCTGCT |
| CD206 reverse | GCGCGTTGTCCATGGTTTCC |
| IL-6 forward | ACTCACCTCTTCAGAACGAATTG |
| IL-6 reverse | CCATCTTTGGAAGGTTCAGGTTG |
| IL-8 forward | TTTTGCCAAGGAGTGCTAAAGA |
| IL-8 reverse | AACCCTCTGCACCCAGTTTTC |
| CSF-1 forward | AGACCTCGTGCCAAATTACATT |
| CSF-1 reverse | AGGTGTCTCATAGAAAGTTCGGA |

Table S1. The primers used for qRT-PCR

| **Immune Infiltrates cells** | **Coef** | **HR** | **95%CI_l** | **95%CI_u** | **P-value** |
| --- | --- | --- | --- | --- | --- |
| CD8+ | -2.705 | 0.067 | 0.008 | 0.561 | 0.013 |
| CD4+ naive | -6.103 | 0.002 | 0 | 189341.9 | 0.512 |
| CD4+ memory activated | -13.458 | 0 | 0 | 1708.398 | 0.207 |
| CD4+ memory resting | -1.917 | 0.147 | 0.032 | 0.677 | 0.014 |
| Tregs | 5.539 | 254.318 | 1.474 | 43880.93 | 0.035 |
| B cell memory | 13.485 | 718908 | 0.001 | 3.470211e+14 | 0.186 |
| Neutrophil | 3.873 | 48.086 | 0.007 | 345804.3 | 0.393 |
| Monocyte | -0.69 | 0.502 | 0.008 | 33.003 | 0.747 |
| Macrophage M0 | 3.262 | 26.102 | 5.455 | 124.902 | <0.001 |
| Macrophage M1 | -0.384 | 0.681 | 0.007 | 70.78 | 0.871 |
| Macrophage M2 | 1.603 | 4.966 | 1.069 | 23.06 | 0.041 |
| Myeloid dendritic cell activated | -38.631 | 0 | 0 | 7.230014e+51 | 0.632 |
| Myeloid dendritic cell resting | 6.764 | 866.378 | 0.326 | 2304583 | 0.093 |
| NK cell activated | -1.251 | 0.286 | 0.003 | 23.464 | 0.578 |
| NK cell resting | 1.007 | 2.737 | 0.003 | 2899.939 | 0.777 |
| Mast cell activated | -2.33 | 0.097 | 0.007 | 1.454 | 0.091 |
| Mast cell resting | 0.335 | 1.398 | 0.001 | 1389.022 | 0.924 |
| Eosinophil | 75.034 | 3.86322e+32 | 0 | 8.610441e+71 | 0.105 |
| T cell follicular helper | 1.392 | 4.024 | 0.03 | 531.699 | 0.576 |
| T cell gamma delta | 1.106 | 3.022 | 0.001 | 7091.122 | 0.78 |

Table S2. Cox proportional hazards model of CIBERSORT in LIHC (n=371)

| Formula | name | r_i_glide_gscore | r_psp_MMGBSA_dG_Bind | r_psp_Lig_Strain_Energy | $CLUSTER |
| --- | --- | --- | --- | --- | --- |
| C44H62N10O10S2 | Deltorphin | -10.284898 | -88.346077 | 9.5739727 | 1 |
| C43H46F3N7O7S | GMB-475 | -8.7072525 | -83.394081 | 12.715555 | 2 |
| C50H56N16O4 | Linagliptin Methyldimer | -9.1693087 | -81.447189 | 10.023511 | 3 |
| C39H33Cl3N2O5S | Ecopladib | -10.923441 | -81.128593 | 12.106576 | 4 |
| C38H44ClN5O3 | BAY-850 | -9.6695747 | -79.394829 | 9.4716654 | 5 |

Table S3. Detailed information of the top five small molecule compound.
